# Does rapid sequence divergence preclude RNA structure conservation in vertebrates?

**DOI:** 10.1093/nar/gkac067

**Published:** 2022-02-21

**Authors:** Stefan E Seemann, Aashiq H Mirza, Claus H Bang-Berthelsen, Christian Garde, Mikkel Christensen-Dalsgaard, Christopher T Workman, Flemming Pociot, Niels Tommerup, Jan Gorodkin, Walter L Ruzzo

**Affiliations:** Center for non-coding RNA in Technology and Health (RTH), University of Copenhagen, Denmark; Department of Veterinary and Animal Sciences, University of Copenhagen, Denmark; Center for non-coding RNA in Technology and Health (RTH), University of Copenhagen, Denmark; Steno Diabetes Center Copenhagen, Gentofte, Denmark; Center for non-coding RNA in Technology and Health (RTH), University of Copenhagen, Denmark; National Food Institute, Technical University of Denmark, Kgs. Lyngby, Denmark; Center for non-coding RNA in Technology and Health (RTH), University of Copenhagen, Denmark; Center for non-coding RNA in Technology and Health (RTH), University of Copenhagen, Denmark; Center for non-coding RNA in Technology and Health (RTH), University of Copenhagen, Denmark; Center for Biological Sequence Analysis, Technical University of Denmark, Denmark; Center for non-coding RNA in Technology and Health (RTH), University of Copenhagen, Denmark; Steno Diabetes Center Copenhagen, Gentofte, Denmark; Center for non-coding RNA in Technology and Health (RTH), University of Copenhagen, Denmark; Department of Cellular and Molecular Medicine (ICMM), University of Copenhagen, Denmark; Center for non-coding RNA in Technology and Health (RTH), University of Copenhagen, Denmark; Department of Veterinary and Animal Sciences, University of Copenhagen, Denmark; Center for non-coding RNA in Technology and Health (RTH), University of Copenhagen, Denmark; Computer Science and Engineering and Genome Sciences, University of Washington, USA; Fred Hutchinson Cancer Research Center, Seattle, USA

## Abstract

Accelerated evolution of any portion of the genome is of significant interest, potentially signaling positive selection of phenotypic traits and adaptation. Accelerated evolution remains understudied for structured RNAs, despite the fact that an RNA’s structure is often key to its function. RNA structures are typically characterized by compensatory (structure-preserving) basepair changes that are unexpected given the underlying sequence variation, i.e., they have evolved through negative selection on structure. We address the question of how fast the primary sequence of an RNA can change through evolution while conserving its structure. Specifically, we consider predicted and known structures in vertebrate genomes. After careful control of false discovery rates, we obtain 13 *de novo* structures (and three known Rfam structures) that we predict to have rapidly evolving sequences—defined as structures where the primary sequences of human and mouse have diverged at least twice as fast (1.5 times for Rfam) as nearby neutrally evolving sequences. Two of the three known structures function in translation inhibition related to infection and immune response. We conclude that rapid sequence divergence does not preclude RNA structure conservation in vertebrates, although these events are relatively rare.

## INTRODUCTION

RNA transcripts are widely studied, usually ignoring their secondary structure. However, their structure is often essential to their function, and a *conserved* structure strongly supports this linkage. There have been several efforts to identify conserved RNA structures, including (among others) those that rely on sequence-based alignments (1,2) and those based on structural alignments (3). Several genome-wide screens for RNA secondary structures conserved in vertebrates have been performed (2,4–8); they predict complementary sets of candidate loci, some of which overlap with annotated noncoding RNAs (ncRNAs). The computational screens look for structures with more (structure-preserving) compensatory basepair changes than expected by chance, i.e. negatively selected secondary structures. The majority of these predicted structures also exhibit negative selection on the primary sequence (8), i.e. the sequence is depleted of substitutions, insertions and deletions during evolution. Some conserved RNA structures, however, lack strong sequence conservation, presumably because evolution predominantly constrains their structure. One extreme example that differs greatly in sequence among vertebrates is the telomerase RNA (9), i.e., the seed alignment of respective Rfam family *Telomerase-vert* (RF00024) has pairwise sequence identity (SI) of 45%. Often, good structural alignments of such families are possible only through hand-curated comparative analyses of covarying basepairs. Furthermore, the lack of sequence conservation has hampered the automated detection of other RNA families with similar characteristics (10). Hence, whether structures with low sequence conservation across vertebrates are common remains an open question.

Unusual evolutionary rates are always interesting, since they both inform our interpretation of ‘usual’ rates and highlight regions that are probably functionally important. Unusual rates for RNA features, especially high mutation rates, are understudied, motivating why our work focuses on exactly this. A popular measure for accelerated evolution based on an alignment and a global model of neutral evolution is the phylogenetic *P*-value (phyloP) (11). phyloP classifies individual alignment sites as conserved or accelerated by comparing to the rate of evolution that is expected under neutral drift. However, mutation rates are linked on a mega-base scale (12) and are sensitive to local covariates such as G+C content, making it desirable to use a more local null model. For this purpose, we have extended the approach used in our previous work (8), which was inspired by the approach of Ponjavic *et al.* (13). In short, as the evolution of virtually all ancestral transposable elements, i.e. ancestral repeats (ARs), present in the last common ancestor of human and mouse has been predominantly neutral (14), we use the evolutionary rates of ARs found near features of interest as our local model of neutral evolution. Furthermore, we have estimated the false discovery rate (FDR) of our test statistic, including the effect of G+C content and length of the sequence being evaluated.

Addressing rapid sequence divergence of conserved RNA structures is challenging since the quality of the structural alignment that serves as input constrains the analysis (15). A well-known pairwise structural alignment strategy is the Sankoff algorithm (16). Due to its time complexity of *O*(*L*^6^), where *L* is the sequence length, approximations or heuristics are used in practice. Here, all of the data that we analyze comes from covariance model (CM)-based multiple sequence alignments. CMs (17) are statistical models of RNA learned from multiple alignments and are widely considered to be among the best available automated tools for this task. Much of our data comes from (8), a study based on the widely used *de novo* RNA motif discovery tool CMfinder (3). It optimizes structural multiple sequence alignments through an expectation maximization-style algorithm leveraging CMs. The rest of our data are Rfam multiple sequence alignments (18), also CM-based, but built from CMs trained on manually curated ‘seed’ alignments.

In short, the remainder of the paper will detail our statistical approach for estimating rapid sequence evolution and for controlling its false discovery rate. Using these tools, we analyze known (Rfam (18)) and *de novo* predicted (8) RNA structures conserved in vertebrates to identify ones that appear to have evolved with very low selection pressure on their sequences despite their structure conservation. We term these as structures with rapidly evolving sequence.

## MATERIALS AND METHODS

### Datasets

For known RNA families, we consulted Rfam version 14.0 (18). Firstly, for all 1510 human seed sequences (753 Rfam families) we retrieved the homologous rhesus macaque (rhemac3) and mouse (mm10) sequences from the human (hg38)-centered 7-way vertebrate UCSC Genome Browser alignment (http://hgdownload.soe.ucsc.edu/goldenPath/hg38/multiz7way/hg38.7way.maf.gz). 940 human seed sequences from 640 Rfam families are partly aligned (≥1 bp) and, hence, anchored to rhesus macaque and mouse. From this, we made global structure alignments of the respective genomic loci with Infernal cmalign (version 1.1.2) (19) guided by the Rfam family covariance model (cmalign -g). For 642 human seed sequences from 414 Rfam families, a covariance-model-guided 3-way alignment showed fewer than 20% of the basepairs in the Rfam consensus structure overlapped by gaps. Finally, we removed columns containing a gap in any of the three sequences and filtered these ‘de-gapped’ 3-way alignments to remove those shorter than 80 columns or that overlapped repeats (RepeatMasker version 4.0.7). After filtering, 255 Rfam families were retained.

For *de novo* predicted structures we chose the Conserved RNA Structure (CRS) resource that searched with CMfinder the human-centered 100-way vertebrate UCSC Genome Browser alignment (8). The resource was filtered to 40078 structures that satisfy the following criteria: FDR of structure prediction less than or equal to 15%, human sequence length ≥100, no overlap to RepeatMasker, conservation in human (hg38), rhesus macaque (rhemac3) and mouse (mm10), and the ‘de-gapped’ 3-way alignment of those species (i.e. after removing columns containing a gap in any of the three) has at least 80 columns. ‘De-gapping’ the 3-way alignments removed a fraction of 0.11±0.11 of alignment columns. Henceforth in this paper, ‘CRS’ means this subset of 40k *de novo* structures (see Supplementary Table S3 for complete list with relevant annotations). Note that the ‘de-gapped’ alignments are only used for evolutionary selection analysis but full sequences are retained for any structure prediction related analysis.

The signals of structure conservation in *de novo* predicted structures were compared to (i) all 2791 Rfam (version 14.0 (18)) seed alignments, (ii) 831 Rfam seed alignments filtered for vertebrate sequences and (iii) 921 CMfinder predicted structure-based alignments representing 547 Rfam families. For the latter we collected the human (hg38) centered 100-way vertebrate UCSC Genome Browser alignments that overlap the 1510 human sequences in the Rfam seed alignments. Then we ran CMfinder (version 0.2.2; pscore calculation uses the UCSC hg38 100-way phylogenetic tree) on these human sequences and their homologous sequences (as well their reverse complementary sequences) to predict structure-based alignments. For 921 human sequences CMfinder predicts at least one alignment with pscore ≥50 (score cutoff used in (8)), and we retrieved the one with maximal length.

### Evolutionary selection analysis

Prior knowledge of nucleotide substitution rates is important to reliably estimate evolutionary distances (20). One such approach is the General Reversible Process substitution model, also known as general time-reversible, or REV/GTR (20) (see Supplementary Methods S1). We use this model, as implemented in the baseml program from the PAML package (version 4.19j (21)), to estimate pairwise distances between corresponding human and mouse primary sequences. Here, as baseml has no model for indels, the sequence distances of structures and ARs are estimated from ‘de-gapped’ alignments (see Datasets).

**Figure 1.**
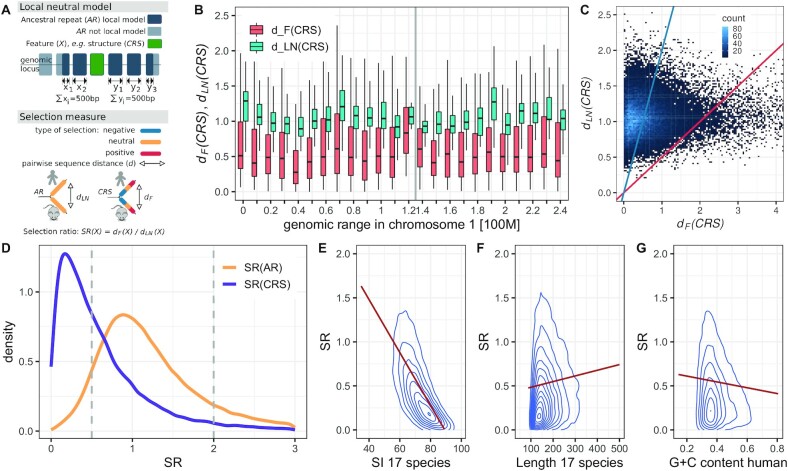
Description of local neutral model and selection ratio’s correlation to covariates. (**A**) The local neutral model is defined by neutrally evolved ancestral repeats (*AR*; blue boxes) that are *local* to a feature (e.g. conserved RNA structure *CRS*; green box). Local (dark blue boxes) are the first 1000 positions of concatenated ARs around a feature. The pairwise sequence distance (*d*) is calculated between human and mouse for both the local neutral model of a CRS (*d*_*LN*_(*CRS*)) and the CRS itself (*d*_*F*_(*CRS*)). The type of selection of features is estimated by the selection ratio (*SR*). (**B**) Distribution of the sequence distance along human chromosome 1 in 100 kb windows for both CRSs (*d*_*F*_(*CRS*)) and their corresponding local null (*d*_*LN*_(*CRS*)) illustrates the linkage of the mutation rates on large scales. Gray vertical line indicates the centromere position. (**C**) Scatterplot of sequence distance of CRSs (*d*_*F*_(*CRS*)) and sequence distance of corresponding local null (*d*_*LN*_(*CRS*)). Points above the blue line are CRSs under negative selection and points below the red line are CRSs with rapidly evolving sequence based on our threshold definition (*SR* < 0.5 and *SR* > 2 respectively). (**D**) The distribution of selection ratio for CRSs (*SR*(*CRS*)) and individual ARs (*SR*(*AR*)). For *SR(AR)* distribution, we show one of the 10 independent samplings of ARs from the *FDR(SR)* calculation. As expected *SR(AR)* is distributed around one (note the color of *SR(AR)* is the same as for neutral selection in panel **A**). Dashed vertical lines mark our thresholds for structures under negative selection (*SR* < 0.5) and structure with rapidly evolving sequence (*SR* > 2). (E–G) Correlation of covariates of conserved structures to *SR(CRS)* is shown as 2d density estimation and linear regression (only *SR* lower than 2). (E, F) are measured from the 17 species structure alignments. The Spearman’s correlation coefficients are (**E**) ρ = −0.76, (**F**) ρ = 0.13 and (**G**) ρ = −0.08.

Ponjavic *et al.* (13) proposed quantifying selection on candidate features by analyzing the pairwise sequence distances between corresponding human and mouse sequences relative to a local neutral model. We have adapted their method as summarized in Figure 1A. A *local* neutral model is needed as the mutation rate of neutrally evolving sequences is not uniform across the genome (12). This is illustrated for human chromosome 1 in Figure 1B (see also Supplementary Figure S4).

Ancestral repeats (ARs) are used as representatives of neutrally evolving sequences. ARs are repeat families present in both human and mouse, hence, phylogenetically ‘ancestral’. Specifically, among all repeats annotated by RepeatMasker (version 4.0.7), we first identify the subset obtained as suggested in Yang *et al.* (22): (i) all non-primate specific repeats, (ii) all non-rodent specific repeats, (iii) all repeats that are not Alu elements and (iv) all repeats that diverged >25%, and L1 elements that diverged >20% from the reconstructed ancestral sequence. Finally, the human, rhesus macaque and mouse sequences of the Yang-defined repeats were collected from human (hg38)-centered 7-way vertebrate UCSC Genome Browser alignments. Our ARs are the ∼1 million repeats with alignments containing all three species, and satisfying the other criteria listed above. The ARs comprise 37% long interspersed nuclear elements (about one quarter of which are L1 elements), 31% short interspersed nuclear elements, 14% long terminal repeat retrotransposons, 14% hAT transposons, and 3% Tc1/marine transposons.

For a feature *X*, i.e. a specific pair of human and mouse sequences such as a CRS (or a specific AR, as a control), our ‘local neutral’ consists of the sequences formed by concatenating the 1000 bases of ‘de-gapped’ ARs nearest to (but not in) *X*, i.e. the nearest 500 bases 5′ to *X* and the nearest 500 bases 3′ to *X*. Letting *d*_*LN*_(*X*) denote the (REV/GTR; see above) distance between these local neutral human and mouse sequences, and letting *d*_*F*_(*X*) be the distance between the human and mouse sequences of the feature *X* itself, the selection ratio *SR* of feature *X* is defined as: $SR(X) = \frac{d_{F}(X)}{d_{LN}(X)}$. For example, *SR* < 1 is an indication that the underlying mutation rate of a feature is lower than expected for neutral drift. Here, we investigate the selection of conserved RNA structure and measure the sequence distance of CRSs (*X* = *CRS*).

In Seemann *et al.* (8), the local neutral model for a given feature consisted of ARs within 1kb of the feature (Supplementary Figure S1). For the current paper, to improve the robustness of the null model, we instead define it to consist of 1000 positions within ARs around a feature (500 bp up- and 500 bp downstream; Figure 1A). This reduces the variance of the *SR* statistic by increasing and freezing the number of AR nucleotides included in each comparison, while retaining sensitivity to locally varying evolutionary rates. We also explored a second alternative. Both had comparable scores (ρ = 0.94; see Supplementary Methods S2 and Supplementary Figure S2 for detailed description and comparison). Based on the *SR* distribution (see Supplementary Figure S3), the chosen model for neutral evolution appears better.

We define a structure with *SR* < 0.5, i.e. one with less than half the local neutral mutation rate, as a structure under negative selection, and a structure with *SR* > 2, i.e. one with twice the neutral mutation rate, as a structure with rapidly evolving sequence. For Rfam families we relaxed the criterion to *SR* > 1.5 (these thresholds seem to highlight a small, confidently identifiable, subset of candidates, but the precise cutoffs are ultimately arbitrary, of course.)

### False discovery rate of selection ratio

We can calculate the *SR* of a specific AR (*X* = *AR*) just as we can for any other feature by comparing its human-mouse sequence distance to that of its neighboring ARs: *SR*(*AR*) = *d*_*F*_(*AR*)/*d*_*LN*_(*AR*). Given our assumption that ARs have evolved neutrally, an AR’s expected *SR* is 1.0. We estimated the rate of false discoveries of extreme *SR* values (*FDR(SR)*) by calculating the *SR* of 120000 randomly sampled ARs of length greater than 80 bp, as described below.

We observed that *SR* is related to the G+C content and length of the *SR* numerator (i.e. feature). Hence, the structures and sampled ARs were divided into ranges of these two covariates: G+C content in human starts with an interval [0–0.25], followed by seven half-open intervals of width 0.05 from (0.25–0.30] to (0.55–0.60], and (0.60–1.00]; *SR* numerator length (0–100],(100–150],(150–200],(200–300],(300–500]. The latter is the number of columns of the ‘de-gapped’ 3-way alignment since the ‘de-gapped’ length is a better predictor of *FDR(SR)*. Then the *FDR(SR)* was estimated for each structure inside its range of G+C content and length as follows. Since we were interested in both the significance of small *SR* (*SR* < 0.5: negative selection) and large *SR* (*SR* > 2: rapidly evolving sequences) the *FDR(SR)* was estimated inside different ranges of *SR*: [0–0.1], 39 half-open intervals of width 0.1 from (0.1–0.2] to (3.9–4.0] and (4.0–∞). For each GC × length × *SR* bin, *FDR(SR)* was calculated as the ratio between the fraction of false positives (sampled ARs) and the fraction of true plus false positives (structures) in that bin. In slightly more detail, for each structure *X* of interest, let *n*_*X*_ be the total number of structures falling in the same bin as *X* based on G+C content and length, and let *t*_*X*_ be the number of them having *SR* in the same bin as *X*’s selection ratio. Let *m*_*X*_ and *s*_*X*_ be the analogous counts among 120000 randomly sampled ARs. Then *X*’s estimated *FDR(SR)* is $\min \left(\frac{s_X/m_X}{t_X/n_X},1\right)$. For example, in a particular GC × length bin, if 1% of the ARs and 5% of structures fall in a given *SR* bin, then we estimate those structure’s *FDR(SR)* to be 0.2. We report the mean of *FDR(SR)* estimates from 10 independent samplings (of 120000 ARs per sample). Structures with *FDR(SR)* ≤ 0.2 were categorized as negatively selected or rapidly evolving according to their selection ratio (see above).

### Gene annotation, structure conservation and experimental support

*De novo* structures were annotated by accessing gene annotations in GENCODE 35 (23) and GeneCards 4.14 (24) at the same human coordinates, with the latter adding ncRNA genes from a wide range of sources.

The conservation of secondary structures was evaluated by the minimum free energy (MFE) based structure conservation index (SCI) (1) and the phylogenetic expectation of RNA structural covariation (R-scape (25)). SCI was computed using RNAalifold (ViennaRNA package version 2.4.1 (26)) on the 17 species alignments of CRSs and all 2791 Rfam seed alignments. As the focus of this study is on vertebrate genomes we filtered vertebrate sequences in Rfam seed alignments, retaining the 831 Rfam families with at least one vertebrate sequence. R-scape (version 1.5.16 (25)) was executed on the 100 species alignment of CRSs, Rfam seed alignments and its vertebrate subset in two different modes: evaluate given structure (parameters ‘-s –GTp –C16’) and evaluate region for conserved structure (default parameters ‘–GTp –CSELECT’). In the first mode R-scape runs two independent covariation tests, one for the basepairs in the consensus structure (predicted or annotated) and the other for all the remaining possible pairs. In the second mode all possible pairs are analyzed equally in one single test. We used R-scape to estimate the number of basepairs with significant covariation support (multiple-test-corrected *E*-value < 0.05) and the statistical power of the alignments. The R-scape model considers both sequence phylogeny and total number of single residue substitutions (27). The alignment power is defined as the fraction of basepairs expected to have significant covariation support, and high-power alignments have >10% power as defined by Rivas *et al.* (27).

The transcriptional activity of *de novo* structures was examined in Seemann *et al.* (8). In short, uniquely mapped reads from publicly available total RNA-seq libraries of 19 tissues (ENCODE phase 3 (28)) and poly(A) RNA-seq libraries of 16 tissues (Illumina Human Body Map 2.0 (29)) were counted that overlap 201 bp windows around the center of CRSs. Read counts were presented as Counts Per Million after cross-experiment Relative Log Expression normalization (CPM/RLE). Matching human and mouse total RNA-seq libraries of four tissues (ENCODE phase 3) were investigated for cross-species comparison. To correct for expression levels of genomic background in a specific library, empirical *P*-values based on the read count distribution of random (not annotated as gene and not repeat masked) genomic loci (201-bp window) were calculated. Here, we report CRSs as expressed if CPM/RLE >1 and *P* <0.01 in at least one tissue, and as putatively expressed if CPM/RLE >0.1 and *P* <0.01. We also checked the atlas of transcriptome-wide RNA secondary structure probing data (30) that collects 58 publicly available structure probing experiments in both human and mouse for available probing signals overlapping *de novo* predicted structures.

## RESULTS

### Estimate of rapid sequence divergence has to be controlled for false positives

Computational tools for predicting conserved RNA secondary structures detect those with more compensatory basepair changes than are expected by the underlying sequence variation, i.e. negatively selected structures (1–3). The genome-wide screen in vertebrates by Seemann *et al.* (8) showed that the majority of predicted structures also exhibit negative selection on the primary sequence. The same study, however, identified a subset of structures that diverged in sequence twice as fast as the ancestral repeats (ARs) within a distance of 1 kb (Supplementary Table S1). Since the mutation rates of ARs vary over the genome in mega-base scale (12) (Figure 1B, Supplementary Figure S4), we decided to recalculate *SR*s with a more robust measure that considers as local neutral model the closest 1000 positions covered by concatenated ARs around structures (Figure 1A). This definition of the null model increased the number of structures, i.e. CRSs, that could be studied compared to Seemann *et al.* (8).

We found 2304 CRSs (6%) with *SR* > 2 (Table 1). However, whereas the 21310 CRSs (53%) with *SR* < 0.5 are clearly more than expected by chance, the same is not valid for structures with high selection ratio (Figure 1C,D). Hence, we need to control for false discoveries by estimating the FDR of the selection ratio, i.e. *FDR(SR)*.

**Table 1.** Statistics of selection ratio (*SR*) at different *FDR(SR)* thresholds for all *de novo* structures, i.e. CRSs, considered in the analysis, candidate CRSs under negative selection (*SR* < 0.5) and candidate CRSs with rapidly evolving sequence (*SR* > 2). $\tilde{x}$ denotes the median. *G+C* is G+C content of human sequence, *SI* is sequence identity between human and mouse, and *Len* is length, in bp, of ‘de-gapped’ human-rhesus macaque-mouse alignment. CRSs with rapidly evolving sequence of 0.2<*FDR(SR)*≤0.33 are listed in the Supplementary Table S2

	All CRSs		*SR* < 0.5		*SR* > 2	
*FDR(SR)*	≤0.2	≤1	≤0.2	≤1	≤0.2	≤1
Number	16.8k	40.1k	16.7k	21.3k	13	2.3k
$\tilde{x}$ (SR)	0.19	0.46	0.19	0.24	3.27	2.94
$\tilde{x}$ (G+C)	0.39	0.38	0.39	0.39	0.44	0.37
$\tilde{x}$ (SI)	0.86	0.77	0.86	0.84	0.57	0.57
$\tilde{x}$ (Len)	140	133	140	136	302	115

FDR methods have been shown to increase power, i.e. correctly rejecting the null hypothesis, by incorporating informative covariates. Therefore we checked for the relationship of *SR* and different structure covariates. As expected, *SR* is negatively correlated to different conservation measures, e.g. sequence identity in the 17 species alignment (Figure 1E; Spearman’s ρ = −0.76), PhastCons from 100 species UCSC alignments (Supplementary Figure S5A; Spearman’s ρ = −0.75), and the species number in the structure alignment (Supplementary Figure S5B; Spearman’s ρ = −0.52). Notably, CRSs of higher estimated false discovery (estimated in Seemann *et al.* (8)) showed a very weak trend towards higher *SR* (Supplementary Figure S5C; Spearman’s ρ = 0.09), which is likely due to the increasing ambiguity of structure-based alignments with decreasing sequence identity, and hence increased number of false positives. Two covariates that are not linked to conservation are alignment length and G+C content with the latter being strongly correlated to RNA structure stability. As both weakly correlate to *SR* (Figure 1F,G; Spearman’s ρ = 0.13, ρ = −0.08, respectively), we corrected for them in the FDR calculation of *SR* (see Materials and Methods).

**Figure 2.**
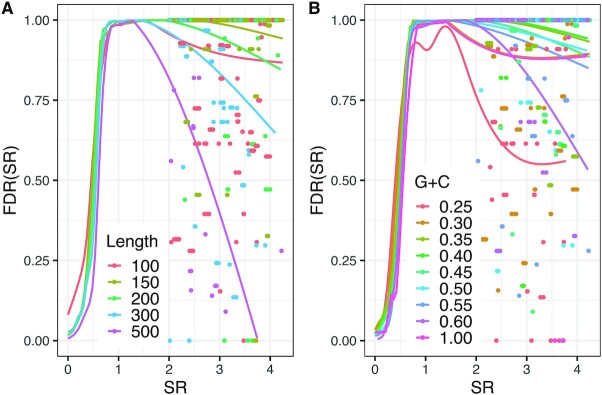
False discovery rate of the selection ratio, i.e. *FDR(SR)*, estimation of *de novo* structures. Structures and sampled ancestral repeats (null model of neutral selection) were divided into ranges of two covariates: ‘de-gapped’ human-rhesus macaque-mouse alignment length [bp] and human G+C content. All pairwise combinations of length and G+C content ranges were applied for *FDR(SR)* estimation. For viewing the impact of the covariates on *FDR(SR)* they are separately viewed. (**A**) Ranges of alignment length [bp] (0–100],(100–150],(150–200],(200–300],(300–500]. (**B**) Ranges of human G+C content [0–0.25], (0.25–0.30], (0.30–0.35], (0.35–0.40], (0.40–0.45], (0.45–0.50], (0.50–0.55], (0.55–0.60], (0.60–1.00]. A generalized additive model (GAM) with restricted maximum likelihood (REML) parameter estimation is fitted to the data in each covariate range. As our focus is on CRSs with rapidly evolving sequence, only CRSs with *SR* > 2 are shown as points (621 CRSs with *SR* > 4.25 are not shown). *FDR(SR)* was estimated inside different ranges of *SR*: 41 half-open intervals of width 0.1 from (0.0–0.1] to (3.9–4.0]. One of the 10 independent samplings of ARs is shown. Supplementary Figure S11 shows the combined plot of both covariates.

Figure 2 shows the impact of the two covariates on the calculation of *FDR(SR)*. For most covariate ranges *de novo* structures under negative selection (*SR* < 0.5) have a low *FDR(SR)* and are, therefore, well distinguishable from the null model. But also some short (length < 150 bp) CRSs seemingly under negative selection are likely to be false positives (Figure 2A). In contrast, the high selection ratio significantly differs from the null model (i.e. have a low *FDR(SR)*) only for a small subset of structures with rapidly evolving sequence. Almost half of the studied structures (16754 out of 40078) have an *FDR(SR)* less than or equal to 20% (Table 1). At this *FDR(SR)* almost all CRSs (16694) are under negative selection and have relatively high sequence conservation (median SI of 86.5%), and only 13 show rapid sequence divergence (median SI of 57%). Instances with low *FDR(SR)* have a longer median alignment length than the average (Table 1), both for structures under negative selection and for those with rapidly evolving sequence. The median G+C content is similar for all *FDR(SR)* thresholds (Figure 2B), but for most CRSs with *SR* >2 and low *FDR(SR)* the G+C content is either low or high. We conclude that the presented computational method for finding structures with rapidly evolving sequence produces a large number of false positive candidates and candidates of low FDR are often longer than 150 bp.

### Known structures with rapidly evolving sequence

The 255 analyzed Rfam families comprise 224 (88%) structures with *SR* < 0.5 and 13 (5%) with *SR* > 1.5. The FDR estimate of the *SR* survey identified 203 structures with negatively selected sequences and three with rapidly evolving sequences (*FDR(SR)* < 0.2). The latter consists of two UTR structures, *IRES Hsp70* (RF00495) and *IFN*γ (RF00259), that regulate the translation of their host mRNA and their sequences are likely to have rapidly evolved in response to changing environmental pathogens affecting the host species as both protein products are involved in bacterial infections and immune responses. The third candidate is the microRNA *mir-657* (RF00988). In the following we discuss these three families in more detail.

The internal ribosome entry site inside of the 5’-UTR of mammalian heat shock protein Hsp70 (*IRES Hsp70*) has diverged almost five times as fast as the local neutral model (selection ratio *SR* = 4.5 with *FDR(SR)* = 0.02). The structure is located in a long and GC-rich locus (G+C content = 67%) of low average pairwise sequence identity (SI = 46%). Rubtsova *et al.* (31) speculate that the relaxed cap-dependence of the 5’-UTR of human Hsp70 mRNA is achieved through the RNA structure, which recruits the ribosome directly to the initiation region surrounding the start codon of Hsp70 (see Silver and Noble (32) for a review). Hsp70 protein chaperones the refolding of heat-denatured peptides to minimize proteolytic degradation as a part of an eukaryotically conserved phenomenon referred to as the heat shock response. As Hsp70 proteins protect cells from high temperature and other forms of stress, e.g. pathogenic bacteria and dietary stress, their pre-translational regulation is likely to be adaptively evolving in vertebrates (33). The human and mouse homologous sequences of the interferon gamma 5’-UTR regulatory element (*IFN*γ) have diverged almost twice as fast as the local neutral model (*SR* = 1.74, *FDR(SR)* = 0.08, G+C content=37%, SI = 59%). The encoded protein is secreted by cells of both the innate and adaptive immune systems and triggers a cellular response to viral and microbial infections. Ben-Asouli *et al.* (34) propose that the RNA structure (specifically a pseudoknot in the structure) inside the 5’-UTR of IFN-γ mRNA locally activates an interferon-inducible protein kinase (PKR) to control the translational yield of its own mRNA. More speculative is the third candidate. The microRNA *mir-657* is intronic of the apoptosis associated tyrosine kinase (AATK) mRNA and has a *SR* of 3.92 (*FDR(SR)* = 0.12, G+C content = 62%, SI = 53%). According to Rfam the microRNA is only conserved in eight primates (full alignment), however, the mouse sequence (mm10/chr11:120014116–120014301) perfectly matches the structure with 79% (co-)varying basepairs between human and mouse (7 covarying and 8 consistent basepairs). Despite the covariance-model-guided 3-way alignment consists of 51% gaps mostly in primates, these gaps are mostly outside of the core hairpin-loop structure. de Faria *et al.* (35) describe a microRNA cluster containing *mir-657* that regulates the proliferation and differentiation of oligodendrocytes precursor cells both in human and mouse. Whereas most microRNAs in the cluster showed similar expression patterns in human and rodents, the expression of *mir-657* was only predicted in human, explaining why a putative function in rodents is unsolved.

### *De novo* structures with rapidly evolving sequence

**Figure 3.**
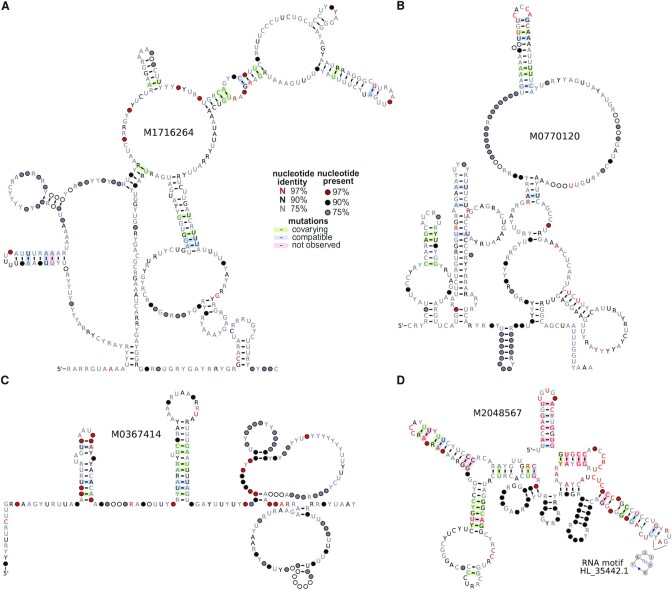
Examples of *de novo* structures with rapidly evolving sequence. Conservation patterns indicated in RNA secondary structures are based on 100 species structure based alignments after removing alignment columns with $\ge 60\%$ of gaps and sequences with $\ge 20\%$ of gaps (drawing by R2R (37)). (**A**) *M1716264* overlaps the long ncRNA lnc-CLEC18B-44 (hg38/chr16:73609195-73609593) and has the following properties: *SR*=2.9, *FDR(SR)*=0.15, GC(human) = 0.36, SI(17 species)=60.2%, Length(17 species) = 542 bp, SCI(17 species) = 0.13. (**B**) *M0770120* overlaps the 3’-UTR of mRNA TIPARP (hg38/chr3:156705568–156705927) and has the following properties: *SR*= 2.9, *FDR(SR)*= 0.15, GC(human) = 0.36, SI(17 species) = 65.4%, Length(17 species) = 387 bp, SCI(17 species) = 0.12. (**C**) *M0367414* is intronic of the long ncRNA LINC00871 (hg38/chr14:45954247–45954488) and has the following properties: *SR*= 3.3, *FDR(SR)*= 0.09, GC(human) = 0.23, SI(17 species) = 51.9%, Length(17 species) = 271 bp, SCI(17 species) = 0.16. (**D**) *M2048567* overlaps the processed pseudogene AC108673.1 (hg38/chr3:129046313–129046527) and has the following properties: *SR*= 3.6, *FDR(SR)*= 0.20, GC(human) = 0.71, SI(17 species) = 64.0%, Length(17 species) = 257 bp, SCI(17 species) = 0.30. The fitted RNA motif HL_35442.1 (36) contains a conserved trans oriented Sugar-Edge Watson–Crick basepair with both isosteric basepairs G–A and A–A occurring in the alignment, and was only found in 2% of randomly selected structures (Supplementary Methods S3).

As the Rfam analysis has shown, the few structures of high *SR* and low *FDR(SR)* are promising candidates of adaptive regulatory function. Hence, we sought for further support of the *de novo* structures. Of the 13 predicted *de novo* structures with rapidly evolving sequence (Table 1 and Supplementary Table S4), two co-localize with ncRNAs (Figure 3A and D), two originate from 3’-UTRs (*ABL1* and *TIPARP*; Figure 3B), eight are intronic (six mRNA and two ncRNA; Figure 3C) including two inside retained introns of protein-coding genes *KDM2B* and *SNRNP200* (putative long ncRNAs), and one is without current annotation. The length of their human sequence ranges from 103 to 399 bp, and they are conserved in 39–79 species from the 100 species tree (median is 56). All candidates are conserved throughout a large fraction of the mammalian subtree, but only the 3’-UTR structure *M1190814* and the ncRNA co-localized structure *M2048567* are also partly conserved in birds and lobe-finned fish. For instance, the structure of CRS *M1956240*, that overlaps a noncoding isoform of the small nuclear ribonucleoprotein U5 subunit 200 (SNRNP200), is well conserved in eutherian animals despite sequence identity of only 56% in the 17 species tree (Supplementary Figure S6A). Additional support for conserved structures comes from known three-dimensional (3D) motifs from the RNA 3D Motif Atlas (36) that fit inside hairpins or interior loops of nine *de novo* structures (Supplementary Methods S3; Supplementary Table S4). Structures with rapidly evolving sequences are not atypical among putative conserved structures w.r.t. the occurrence of 3D motifs (Supplementary Figure S7), which is in line with the expectations. For instance, the third hairpin loop of CRS *M2048567* matches the motif *HL_35442.1* (Figure 3D).

#### Experimental support

Only one CRS out of 13, i.e. *M0665102*, is without any expression support based on the investigated publicly available expression data (see Methods and Supplementary Table S4). In addition to the structures co-localized with retained intronic regions and UTRs, this data supports the transcriptional activity in human of three intronic structures (*M0906221*, *M0989211*, *M1493678*) and one ncRNA (*M2048567*) (Supplementary Table S4). For instance, CRS *M1493678* is located in the middle of a 10kb long intron of neuron navigator 2 (NAV2) and overlaps a distal enhancer-like signature (ENCODE identifier EH38E1526134 (38)). Total RNA-seq experiments show consistently its expression in the human and mouse hindbrain (cerebellum) but not in the forebrain (diencephalon). Also intronic *M0367414* and *M0785346*, ncRNA co-localized *M1716264*, and intergenic *M1242792* show weak signatures of expression in at least one human tissue. The RNA structure probing database RASP (30) contains a wide-range of transcriptome-wide structure probing experiments. RASP supports a highly structured 3’-UTR of ABL1 in both human and mouse overlapping the CRS *M1190814*. However, for the other 12 candidates none of the structure probing experiments cover their genomic loci (no expression in the experimental setup), hence, the structure probing data neither supports nor refutes the *de novo* structures. As structure probing has low sensitivity for weakly expressed genes (39), it is not surprising that structure probing data is missing for the primarily noncoding structure candidates.

#### Structure conservation

**Figure 4.**
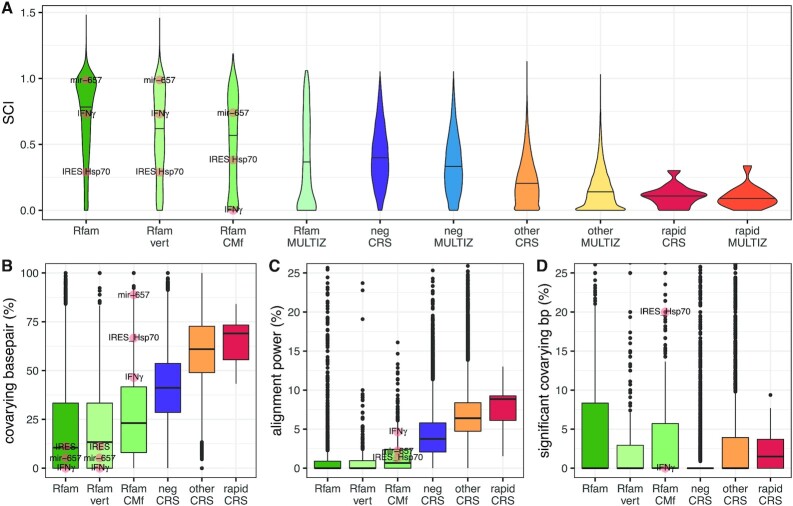
Signals of structure conservation in *de novo* and known secondary structures. (**A**) Structure conservation index (SCI) calculates the consistency between the structures of the individual sequences and the consensus structure in terms of minimum free energy (MFE). (**B**) Fraction of covarying basepairs in the annotated consensus structure. (**C**) Alignment power is the fraction of basepairs expected to show a significant covariation signal as calculated by R-scape. (**D**) Fraction of basepairs that show a significant covariation signal in the two-set statistical test (one test for annotated basepairs (bp), another for all other pairs) by R-scape (*E* < 0.05). We distinguish *de novo* structures with rapidly evolving sequence (*rapid CRS*: *SR* > 2 and *FDR(SR)*≤0.2), under negative selection (*neg CRS*: *SR* < 0.5 and *FDR(SR)*≤0.2), and other (*other CRS*). For comparison, Rfam (version 14.0) seed alignments (*Rfam*), their subset of vertebrate sequences (*Rfam vert*), and CMfinder predicted structure-based alignments of the human sequences in Rfam seed alignments and their homologous sequences extracted from the human (hg38) centered 100-way vertebrate MULTIZ alignments (*Rfam CMf*) were analyzed. The SCI in (A) has also been calculated for human (hg18) centered 17-way vertebrate UCSC Genome Browser alignments (MULTIZ) overlapping the human sequence of CRSs, and the human (hg38) centered 100-way MULTIZ overlapping the human sequences in Rfam seed alignments, illustrating the improved structure conservation signal in the structure-based alignments of CRSs. In (A) and (B) all 2,791 Rfam seed alignments and 831 vertebrate alignments are shown, whereas in (**C**) and (**D**) R-scape analyzed only 1966 seed alignments and 712 vertebrate alignments as for the others (including *mir-657*) the covariation in the alignment is too small (mostly due to too few sequences). The Rfam families *IRES Hsp70* (RF00495), *IFN*γ (RF00259) and *mir-657* (RF00988) with rapidly evolving sequence are indicated. If not then their values are zero, e.g. *R*-scape estimates expected and observed significantly covarying basepairs to be zero in the *Rfam* and *Rfam vert* alignments for all three families. *mir-657* has $56\%$ significant covarying bps (5 out of 9 bp) and, hence, is out of y-axis limits in (D). The median values are marked as horizontal lines. All three Rfam families with rapidly evolving sequence have exclusively vertebrate sequences in their seed alignments, hence *Rfam* and *Rfam vert* values are the same for them: *IRES Hsp70* – 12 sequences from primates and 2 from cattle (see Supplementary Figure S8), *IFN*γ – 4 from primates and 1 from cattle, and *mir-657* – 2 from primates.

The structure conservation index (SCI) measures the conservation of the MFE structure in an alignment (1). In general, SCI is negatively correlated to the *SR* measure (Supplementary Figure S5D; Spearman’s ρ = −0.47). *De novo* structures under negative selection have a high SCI that is comparable to the SCI distribution of known structures that were realigned with CMfinder (labels ‘neg CRS’ and ‘Rfam MULTIZ’ in Figure 4A). The remaining CRSs tend to lower SCIs. The three CRSs with rapidly evolving sequence that have the strongest structure conservation based on SCI are shown in Figure 3C and D and Supplementary Figure S6B. The latter (*M0906221*) is intronic of the mRNA CAMK4 which is implicated in transcriptional regulation in lymphocytes, neurons and male germ cells, with the acquisition of the latter being linked to enhanced evolvability (40). Also the Rfam family with highest sequence divergence between human and mouse, *IRES Hsp70*, has an SCI comparable to the *de novo* structures with rapidly evolving sequence (see Rfam seed and CMfinder alignment in Supplementary Figure S8). However, as the sequence composition has large impact on the free energy of a structure, the MFE-based SCI measure may not capture the structure conservation in structure-based alignments with rapidly evolving sequence. If the homologs of a conserved RNA structure have different G+C contents then their free energies will vary despite a conserved RNA structure which will lead to low SCI. We also calculated SCI for the genome-wide sequence-based alignments (vertebrate UCSC Genome Browser alignments) that overlap the human sequence of CRSs and Rfam seeds, and observed, as expected, lower SCI compared to the structure-based alignments (Figure 4A).

Conservation of RNA structures induces pairwise covariations in sequence alignments. We explored the rate of covariation in known and *de novo* structure-based 100 species alignments (Figure 4B–D). On average, *de novo* predicted structures with rapidly evolving sequence have the highest fraction of covarying basepairs whereas the Rfam seed alignments contain the lowest fraction (Figure 4B). Here, a basepair is counted as covarying if at least two canonical basepairs of compensatory substitutions exist in the respective alignment columns, e.g. G:C ↔ A:U ↔ U:A ↔ C:G. Depending on the alignment properties (e.g. number of species and their phylogentic distances) different structural covariation is expected. In Figure 4C we estimated the alignment power (fraction of basepairs expected to show a significant covariation signal) with R-scape (27), and found that the percentage of covarying basepairs in the different investigated classes of structure alignments follows our expectations. On average no significant covarying basepair (E<0.05) was detected in any of the alignments by using R-scape’s default statistical test that equally tests all possible basepairs (Supplementary Figure S9). In case the structure is known *a priori* the two-set covariance test of R-scape is, however, more sensitive. It compares the basepairs in the consensus structure with all other possible pairs. Using the two-set covariance test we estimated that at the third quartile Rfam seed alignments have 8% significant covarying basepairs which is twice as high than for the *de novo* structures (Figure 4D). However, as the latter are conserved throughout vertebrates they have to be compared to the Rfam alignments restricted to vertebrate sequences. Indeed, the percentage of significant covarying basepairs in alignments of vertebrates are comparable. Note that the set of negatively selected CRSs also contains many conserved structures of high sequence identity which is why the majority has no significant covarying basepairs. The seed alignments of the three Rfam families *IRES Hsp70*, *IFN*γ and *mir-657* with rapidly evolving sequences have zero expected and observed covarying basepairs which may be, at least partly, explained by only 14, 5 and 2 sequences (all vertebrates) in their seed alignment, respectively. In general, we see less compensatory basepair changes in known compared to *de novo* structures (Figure 4B-D). In addition, hand-curated Rfam seed alignments have less covariance signals than computational structure-based alignments (predicted with CMfinder) for both known and *de novo* structures.

For the investigated classes of structure alignments, the median of basepairs that significantly covary is only different from zero for the *de novo* structures with rapidly evolving sequence (median = 1.5; Figure 4D), supporting the faster fixation of basepair changes in rapidly evolving sequences. The consensus structure of eight CRSs with rapidly evolving sequence contains at least one significant covarying basepair (*E* < 0.05; Supplementary Table S3), and the consensus structures of two CRSs, *M0367414* (Figure 3C) and *M1242792*, have more covarying basepairs than expected for the phylogenetic correlations and base composition in their 100 species alignments (25). Of the two *de novo* structures with high-power alignments, the consensus structure of CRS *M1493678* is supported by one significant covarying basepair according to the two-set test. The high-power alignment of *M0785346* lacks, however, supporting covariations which may argue against a conserved structure (27).

## DISCUSSION

Starting from known and *de novo* predicted RNA secondary structures, we investigated the subset with rapidly evolving sequence in vertebrates. Vertebrate protein-coding genes of rapidly evolving sequence are rare and occur, for example, in the immune system and in egg/sperm recognition (41). Similar evolution in ncRNAs and especially in RNA structures has not been extensively studied. We show that in silico screens for conserved RNA structures can identify candidate structures with rapidly evolving sequences above the noise level, although they also seem to be rare. Specifically, we predict three known Rfam structures and 13 *de novo* structures with rapidly evolving human and mouse sequences with an estimated *FDR(SR)* of at most 20%.

Differentiating rapid evolution of functional sequences, and especially positive selection, from relaxed genomic constraint is a common issue in selection studies (41). Our definition of the selection ratio *SR* statistic and careful control of its FDR address this issue in our context, by directly assessing the rate of evolution of a specific feature to a locally-calibrated neutral rate. Our calculation of *SR* was inspired by the method of Ponjavic *et al.* (13), but deviates from it in the definition of the local neutral model: whereas Ponjavic *et al.* always compares a candidate feature of length *n* to *n* positions within nearby ARs, we have chosen to always compare to 1,000 positions within nearby ARs, independent of *n*. This change in methodology was motivated by the need to control FDR. Our CRSs are typically much shorter than those studied in Ponjavic *et al.* and ratio statistics such as *SR* can be inflated by chance small denominators at least as easily as by chance large numerators. Setting the sequence length defining the denominator to a fixed and moderately large value reduced our apparent FDR and made it less sensitive to the length of the feature being evaluated. For our purposes, we believe this to be an improvement over Ponjavic *et al.*, and over the variant of it that we used in Seemann *et al.* (8). Supplementary Figure S1 and its accompanying discussion illustrates this.

We also attempted to gain theoretical insight into the factors that influence the FDR of *SR*, i.e. *FDR(SR)*. In particular, assuming that neutral sequences of particular lengths acquire substitutions independently with some fixed probability *P* per position, the *SR* is a ratio determined by substitution counts, each following a binomial distribution with $P\approx1-\mathit {SI}$. With ‘evolutionary distance’ measured either by those counts or by the Jukes-Cantor distance based on them, it is not difficult to numerically calculate FDR for $\mathit {SR}>2$. These calculations clearly reveal dependence of *FDR(SR)* on lengths, sequence identity, and use of counts versus the more realistic Jukes-Cantor model, all of which helped inform our choice of methods. One surprise, however, was that the empirically estimated FDRs described in Methods were sharply higher than those calculated using these simple models. Whether the under-performance of the theoretical models can be addressed by using a richer theory such as the general time reversible model from baseml, or whether it reflects deeper heterogeneities in real genomes remains an open question, but in either case our empirically derived *FDR(SR)* estimates seem to be a conservative choice.

Instead of binning to address the G+C content and length confounder of the FDR estimation, we also considered regression models. For the presented data, however, the within-bin variability in effect size often exceeds the change in mean effect size between adjacent bins, so we do not believe the additional smoothing afforded by a regression-based approach would qualitatively change our results. Furthermore, the confounders’ effects are nonlinear, and we preferred to avoid the extra complexity to fit such models. In addition, binning is simple, easily understood, and has a completely transparent set of parameters (bin boundaries).

The main limitation of our FDR-controlled *SR* measure is its low power for short alignments, as their estimated *SR*s are indistinguishable from randomly selected ARs of similar G+C content and length. For instance, Rfam contains eight additional microRNAs and one snoRNA with high selection ratio, but these relatively short RNA families also have high *FDR(SR)*’s. In addition, short structures under negative selection also tend to have a high *FDR(SR)*.

R-scape estimates that *de novo* predicted structures with rapidly evolving sequence have, on average, at least one significant covarying basepair in the consensus structure. In contrast, the corresponding analysis of Rfam families with rapidly evolving sequence found zero. This is somewhat unexpected, but that analysis was constrained by the small number of sequences in their seed alignments; the picture may change as the families are expanded. We also observe that hand-curated alignments, i.e. Rfam seed alignments, may under-estimate covarying basepairs, whereas computationally predicted structure-based alignments, i.e. CMfinder alignments, may over-estimate them (see Figure 4). As our analysis is sensitive to sequence distances it may impact the prediction of features with rapidly evolving sequences.

A special case of rapid sequence divergence are lineage specific structures as is the case for Human Accelerated Region 1 (42). These are highly conserved except for a single species or phylum where the structure (and sequence) has evolved independently. Lineage specific structures can be predicted with the Selection on Secondary Structure test (SSS-test (43)) that examines the impact of substitutions (using RNAsnp (44)) and indels on RNA structures. The SSS-test complements our approach that finds accumulated mutation rates in conserved structures. Using as input the human-rhesus macaque-mouse alignment of CRSs (including gap columns), SSS-test version 1.0 predicts 27 putative human lineage specific structures (see Supplementary Methods S4 and Supplementary Table S5 for filtering criteria and annotation). However, only one of them matches our definition of a rapidly evolving sequence (but with *FDR(SR)* > 0.2). Two other candidates overlap the human pseudogene TAB3P1 (Supplementary Figure S10). The genomic locus of TAB3 was duplicated and translocated from chromosome X to Y more recently than the human-mouse divergence and presumably had been under negative selection until the duplication event. In the investigated human-centered 100-way vertebrate UCSC Genome Browser alignment the human pseudogene TAB3P1 is aligned to chromosome Y in only two other primates (chimp and green monkey). Instead, the genome-wide alignment has aligned the pseudogene to its parent gene TAB3 on chromosome X in all other primates and some of the other vertebrates. We checked the pairwise alignments of the latest primate assemblies and found that, indeed, the sequence of the pseudogene on chromosome Y was missing in most of the previous primate assemblies including rhesus macaque. We infer that these conserved RNA structures are not lineage specific and, although now probably evolving neutrally in primates, have not been doing so for long enough to erase the signs of negative selection. The example illustrates the constraints of selection analysis on the quality of genome-wide alignments.

In conclusion, our study demonstrates that conserved RNA structures with rapidly evolving sequence are rare events based on the available conservation data. More candidates in non-syntenic genomic regions or of very low sequence conservation may be, however, undiscovered (45) as the investigated *de novo* predicted conserved structures were constrained by genome-wide sequence-based alignments.

## LIST OF ABBREVIATIONS

dAR: ancestral repeat; CRS: subset of conserved RNA structure predicted by Seemann *et al.* (8); FDR: false discovery rate of conserved RNA structure prediction, also from (8); FDR(SR): false discovery rate of the selection ratio; SCI: structure conservation index as defined by Washietl *et al.* (1); SR: selection ratio; SI: average pairwise sequence identity

## DATA AVAILABILITY

Data discussed in this paper are provided in Supplementary Tables S3 to S5 and on https://rth.dk/resources/rnannotator/crs/vert/pages/rapid-evolving-sequences.php.

## Supplementary Material

gkac067_Supplemental_Files
