# Supplementary material for: Does rapid sequence divergence preclude RNA structure conservation in vertebrates?
Source: Nucleic Acids Res. 2022 Feb 21;50(5):2452–63. doi: 10.1093/nar/gkac067 (PMC8934657; doi:10.1093/nar/gkac067)
Supplement: gkac067_Supplemental_Files [file gkac067_supplemental_files.zip › Supplementary_Material.pdf]

Supplementary Materials

Does rapid sequence divergence preclude RNA structure conservation in vertebrates?

Stefan E. Seemann, Aashiq H. Mirza, Claus H. Bang-Berthelsen, Christian Garde, Mikkel Christensen-Dalsgaard, Christopher T. Workman, Flemming Pociot, Niels Tommerup, Jan Gorodkin, Walter L. Ruzzo

Content

1. **Supplementary Methods S1:** Pairwise distance estimation with General Reversible Process substitution model

2. **Supplementary Methods S2:** Comparison of different local neutral models for selection ratio calculation.

3. **Supplementary Methods S3:** RNA 3D motifs.

4. **Supplementary Methods S4:** *De novo* conserved RNA structures with human lineage specific structures.

5. **Supplementary Table S1:** Summary of structures with rapidly evolving sequence in the selection analysis performed by Seemann *et al.* [1].

6. **Supplementary Table S2:** Statistics of selection ratio (*SR*) at different *FDR(SR)* thresholds for all *de novo* structures (complement to Table 1) including alternative local neutral model and candidates with FDR(SR)$\leq$ 0.33.

7. **Supplementary Table S3 (Separate TSV file):** Supportive information of all 40078 conserved RNA structures used in this study.

8. **Supplementary Table S4 (Separate TSV file):** Supportive information of 13 *de novo* conserved RNA structures with rapidly evolving sequence.
   https://rth.dk/resources/rnannotator/crs/vert/v2.1/pages/cmf.data.collection.crsreg.php?crsreg=M0269720,M0367414,M0665102,M0770120,M0785346,M0906221,M0989211,M1190814,M1242792,M1493678,M1716264,M1956240,M2048567

9. **Supplementary Table S5 (Separate TSV file):** Supportive information of 31 conserved RNA structures with putative human lineage specific structures.
   https://rth.dk/resources/rnannotator/crs/vert/pages/cmf.data.collection.crsreg.php?crsreg=M0046445,M0049429,M0297292,M0325303,M0372316,M0377357,M0377358,M0660869,M0776320,M0908557,M1043544,M1206805,M1250162,M1269253,M1279394,M1358254,M1358809,M1513525,M1660801,M1660802,M1942819,M2185801,M2185802,M2432844,M2446369,M2571948,M2572215,M2572500,M2572586,M2573093,M2037356

10. **Supplementary Figure S1:** Schematic description of three different local neutral models.

11. **Supplementary Figure S2:** Scatter plot of selection ratios *SR*s of CRSs with both FDR(*SR(1k)*) and FDR(*SR(100k)*).

12. **Supplementary Figure S3:** The distribution of selection ratio *SR* for two types of local neutral models.

13. **Supplementary Figure S4:** Human-mouse nucleotide distance of ancestral repeats (ARs) in four different human chromosomes.

14. **Supplementary Figure S5:** Correlation between selection ratio *SR* and different features of CRSs.

15. **Supplementary Figure S6:** Examples of *de novo* structures with rapidly evolving sequence.

16. **Supplementary Figure S7:** Observed and expected counts of **A** internal and **B** hairpin loop RNA 3D motifs.

17. **Supplementary Figure S8:** Rfam seed and CMfinder predicted alignment of *IRES Hsp70*.

18. **Supplementary Figure S9:** Fraction of basepairs that show a significant covariation signal in the one-set statistical test (all pairs are tested as equivalent) by R-scape (E<0.05).

19. **Supplementary Figure S10:** Human lineage specific CRS *M1279394*.

20. **Supplementary Figure S11:** False discovery rate of the selection ratio, *i.e. FDR(SR)*, estimation of *de novo* structures.

21. **References for Supplement**

Supplementary Methods S1: Pairwise distance estimation with General Reversible Process substitution model

The pairwise sequence distance d is estimated by using the General Reversible Process / general time-reversible (REV/GTR) model:

$$REV/GTR = \begin{pmatrix} . & \alpha\ \pi_G & \beta\ \pi_C & \gamma\ \pi_T \\ \alpha\ \pi_A & . & \delta\ \pi_C & \varepsilon\ \pi_T \\ \beta\ \pi_A & \delta\ \pi_G & . & \eta\ \pi_T \\ \gamma\ \pi_A & \varepsilon\ \pi_G & \eta\ \pi_C & . \end{pmatrix} \tag{1}$$

This substitution model maintains "detailed balance" so that, for instance, the probability of starting at A and ending at T in evolution is the same as the probability of starting at T and ending at A.

The parameters are adjusted (standardized) so that one unit of time is the time in which we expect to see one change per base:

$$2\ \pi_A\ \pi_G\ \alpha + 2\ \pi_A\ \pi_C\ \beta + 2\ \pi_A\ \pi_T\ \gamma + 2\ \pi_G\ \pi_C\ \delta + 2\ \pi_G\ \pi_T\ \varepsilon + 2\ \pi_C\ \pi_T\ \eta = 1 \tag{2}$$

The parameters are determined from the input alignment.

Based on equation (1) and (2) the rate matrix is:

$$A = \begin{pmatrix} -(\alpha\ \pi_G + \beta\ \pi_C + \gamma\ \pi_T) & \alpha\ \pi_A & \beta\ \pi_A & \gamma\ \pi_A \\ \alpha\ \pi_G & -(\alpha\ \pi_A + \delta\ \pi_C + \varepsilon\ \pi_T) & \delta\ \pi_G & \varepsilon\ \pi_G \\ \beta\ \pi_C & \delta\ \pi_C & -(\beta\ \pi_A + \delta\ \pi_G + \eta\ \pi_T) & \eta\ \pi_C \\ \gamma\ \pi_T & \varepsilon\ \pi_T & \eta\ \pi_T & -(\gamma\ \pi_A + \varepsilon\ \pi_G + \eta\ \pi_C) \end{pmatrix}$$

The transition probability matrix P can be computed for any branch length t from the rate matrix A by matrix exponentiation:

$$P(t) = e^{At}$$

As there is no convenient formula for the elements of *P(t)*, *P(t)* is calculated numerically involving numerical determination of eigenvalues and eigenvectors of associated matrices. This process is described in detail in [2].

The pairwise sequence distance from the GTR model is estimated by finding the value of t for a pairwise alignment S that maximizes the following likelihood:

$$\arg\max\ L(t; S) = \prod_{i \in S} \pi_{n_i}\ P_{m_i\ n_i}(t),$$

where the base i is m_i in one sequence of alignment S and n_i in the other.

We have estimated the parameters and the pairwise sequence distance with the `baseml` program from the PAML package (version 4.19j [3]; `http://abacus.gene.ucl.ac.uk/software/pamlDOC.pdf`).

Supplementary Methods S2: Comparison of different local neutral models for selection ratio calculation

An alternative measure to *SR* described in the main text (here termed as *SR(1k)*) considers all ARs located in a window of 100 kb around CRS (50 kb up- and 50 kb downstream of CRS) and we termed it as *SR(100k)* (Supplementary Figure S1). The selection ratio *SR(100k)* was calculated for each CRS as the median of ratios of estimated base distance of the CRS (d_{CRS}) and the equivalent base distance within local ARs (d_{AR}):

$SR(100k) = \text{Median}(d_{CRS}/d_{AR}\ \forall \text{local ARs})$.

The correlation between *SR(1k)* and *SR(100k)* for CRSs with both FDR(SR)$\leq$ 0.2 is very high (Pearson's correlation coefficient ρ=0.94, Supplementary Figure S2). The *SR* distribution of 120,000 ARs that were used for *FDR(SR)* calculation, should be centered at 1.0 as our approach relies on the hypothesis that ARs have neutrally evolved. This is valid for both tested local neutral models (Supplementary Figure S3). However, both models result in a considerable large number of ARs with extreme *SR*'s. For instance for one of the 10 independent samplings of ARs we estimated *SR(1k)*<0.5 for 7,925 ARs, *SR(100kb)*<0.5: 10,223, *SR(1k)*>2: 17,449, and *SR(100kb)*>2: 11,842. In the main text we present only *SR(1k)*. For completeness we also report the union of both measures in Supplementary Table S2.

Supplementary Methods S3: RNA 3D motifs

An independent structure feature of RNA secondary structures is the existence of modular 3D motifs. These recurrent motifs are found in a variety of non-homologous locations in diverse RNA molecules, and some play architectural roles or provide binding sites for proteins or ligands [4]. Known three-dimensional (3D) motifs from the RNA 3D Motif Atlas [5] were fitted into loop sequences of *de novo* structures. Firstly, the consensus secondary structures were parsed for internal loops and hairpin loops using a custom script. Secondly, for all loops the corresponding columns were cut from the 17 species alignment and, thirdly, these alignments were scored with JAR3D [6] against all motif groups of either internal or hairpin loops from the RNA 3D Motif Atlas (version 3.2). Fitted 3D motifs were filtered for recommended thresholds of JAR3D scores: %passedCutoff>70 (percentage of sequences in the alignment falling into the acceptance region of the motif group that is consistent with the geometry and base pairing of that group) and meanCutoffScore>40 (combined score of alignment score deficit and minimum interior edit distance). To estimate the expected counts of internal and hairpin loop RNA 3D motifs we grouped CRSs into covariate bins of similar G+C content in human, length of the 17 species alignment, and average pairwise sequence identity of the 17 species alignment. For the latter covariate, we used the following bins (in percentage): (0–55],(55–65],(65–75],(75–85],(85–100], and for the other two covariates we used the same ranges as described above. From each of the 36 covariate bins with the same sequence identity as the query CRSs, we randomly selected five structures (totaling 180). Then we ran JAR3D, filtered the results as described above, and compared motifs in CRSs with rapidly evolving sequence with motifs in these 180 random CRSs.

Supplementary Methods S4: *De novo* conserved RNA structures with human lineage specific structures

The SSS-test [7] finds lineage specific positive selection of RNA secondary structure in a set of well-conserved sequences. By default it reports candidates with an estimated FDR of less then 0.3. The criteria of low family diversity used in Walter Costa *et al.* [7] is different from the criteria of CRSs with rapidly evolving sequence, hence, the parameters used in Walter Costa *et al.* [7] (family diversity$\leq$ 10 and SSS-score$\geq$10.0) are not directly applicable on the data in this study. Here, we defined CRSs with SSS-score$\geq$10 in human and SSS-score$<$10 in macaque and mouse and fewer nucleotide changes in macaque and mouse to the consensus sequence than in human as human accelerated region with positively selected structure. The CRSs comprise 27 putative human lineage specific structures (31 CRSs) that were detected with `SSS-test` (Supplementary Table S5). These structured regions are colocalized with 12 putative lncRNAs, 5 pseudogenes, 5 introns (3 mRNAs and 2 ncRNAs), and 2 3'-UTRs. For instance, the CRSs *M1279394* and *M2571948* are located at the 5' end of the TGF-Beta Activated Kinase 1/MAP3K7 Binding Protein 3 Pseudogene 1 (*TAB3P1*, in GeneCards annotated as lncRNA *lnc-PNRC1-2*) on chromosome Y and is highly conserved in sequence and structure from primates to aves. The human sequence has, however, a large number of substitutions of which many are not compatible with the consensus secondary structure (Supplementary Figure S10). CRS *M2037356* colocalizes with the copy of MAT2A_C that overlaps the lncRNA *lnc-SLC35A5-3*. It is almost perfectly conserved in 12 species out of the 17 species tree, but has two compensatory and one disruptive basepair changes in human. MAT2A_C is one out of 6 conserved hairpin motifs that exist in a cluster both in the 3'-UTR of the MAT2A mRNA transcript (MAT2A_A to MAT2A_F), and in the aforementioned locus on chromosome 3 (Rfam annotation).

SR>2<br>all CRSs	SI<40	SI<50	SI<60
FDR≤1	64<br>96	475<br>1004	1323<br>4797
FDR<0.15	33<br>48	244<br>518	736<br>2786
FDR<0.1	14<br>15	73<br>156	241<br>996

Table S1: CRSs with rapidly evolving sequence in the selection analysis performed by Seemann *et al.* [1]. Each cell reports the count of all CRSs identified in the analysis (lower left) and the number thereof that were classed as CRSs with rapidly evolving sequence (*SR*>2; upper right) for different combinations of thresholds for False Discovery Rate (FDR) of CRSs and their average pairwise sequence identity (SI). Categories are inclusive; *e.g.*, the 244 candidate CRSs with rapidly evolving sequence identified with SI<50 and FDR<0.15 include the 33 identified with that FDR threshold and SI<40. Note that the *SR* calculation used in [1] differs from the calculation used in this study.

	all CRSs					SR>2				
	SR(1k)	*SR(100k)*	*SR(1k)* ∪ *SR(100k)*			*SR(1k)*	*SR(100k)*	*SR(1k)* ∪ *SR(100k)*		
FDR(SR)	≤0.2	≤0.2	≤0.2	≤0.33	≤ 1	≤0.2	≤0.2	≤ 0.2	≤0.33	≤ 1
N	16754	17114	18472	20935	40108	13	18	26	44	2399
Median SR	0.19	0.17	0.21	0.24	0.48	3.27	2.82	3.33	3.30	2.91
Min SR	0.00	0.00	0.00	0.00	0.00	2.42	2.30	3.12	2.40	2.00
Max SR	5.11	10.59	16.24	16.24	263551	5.11	10.59	5.11	16.24	263551
Median GC	0.39	0.40	0.40	0.39	0.38	0.44	0.45	0.44	0.30	0.37
Min GC	0.18	0.18	0.18	0.18	0.14	0.21	0.23	0.21	0.21	0.15
Max GC	0.80	0.80	0.80	0.80	0.81	0.71	0.66	0.71	0.71	0.75
Median SI	0.86	0.86	0.86	0.85	0.77	0.57	0.53	0.57	0.57	0.57
Min SI	0.48	0.43	0.43	0.43	0.26	0.48	0.43	0.48	0.43	0.26
Max SI	1.00	1.00	1.00	1.00	1.00	0.68	0.58	0.68	0.68	0.75
Median Len	140	140	140	138	133	302	213.5	302	209	115.5
Min Len	80	80	80	80	80	86	86	86	85	80
Max Len	486	486	486	486	486	348	364	348	368	368

Table S2: Statistics of *SR* at different *FDR(SR)* thresholds of all CRSs considered in the analysis and candidate CRSs with rapidly evolving sequence (*SR*>2). Both tested local neutral models and their union (at least one measure must fulfill the *FDR(SR)* threshold) are shown. *GC* is G+C content of human sequence, *SI* is average pairwise sequence identity between human and mouse, and *Len* is length of human-mouse-macaque alignment after removal of gap columns. Twelve additional CRSs have a *FDR(SR)*> 0.2 but ≤ 0.33 in the local neutral model *SR*(1*k*). They can be found at https://rth.dk/resources/rnannotator/crs/vert/v2.1/pages/cmf.data.collection.crsreg.php?crsreg=M0269721,M0335095,M0341484,M0902417,M1004485,M1283457,M1419778,M1996186,M2068266,M2200901,M2331224,M2347054.

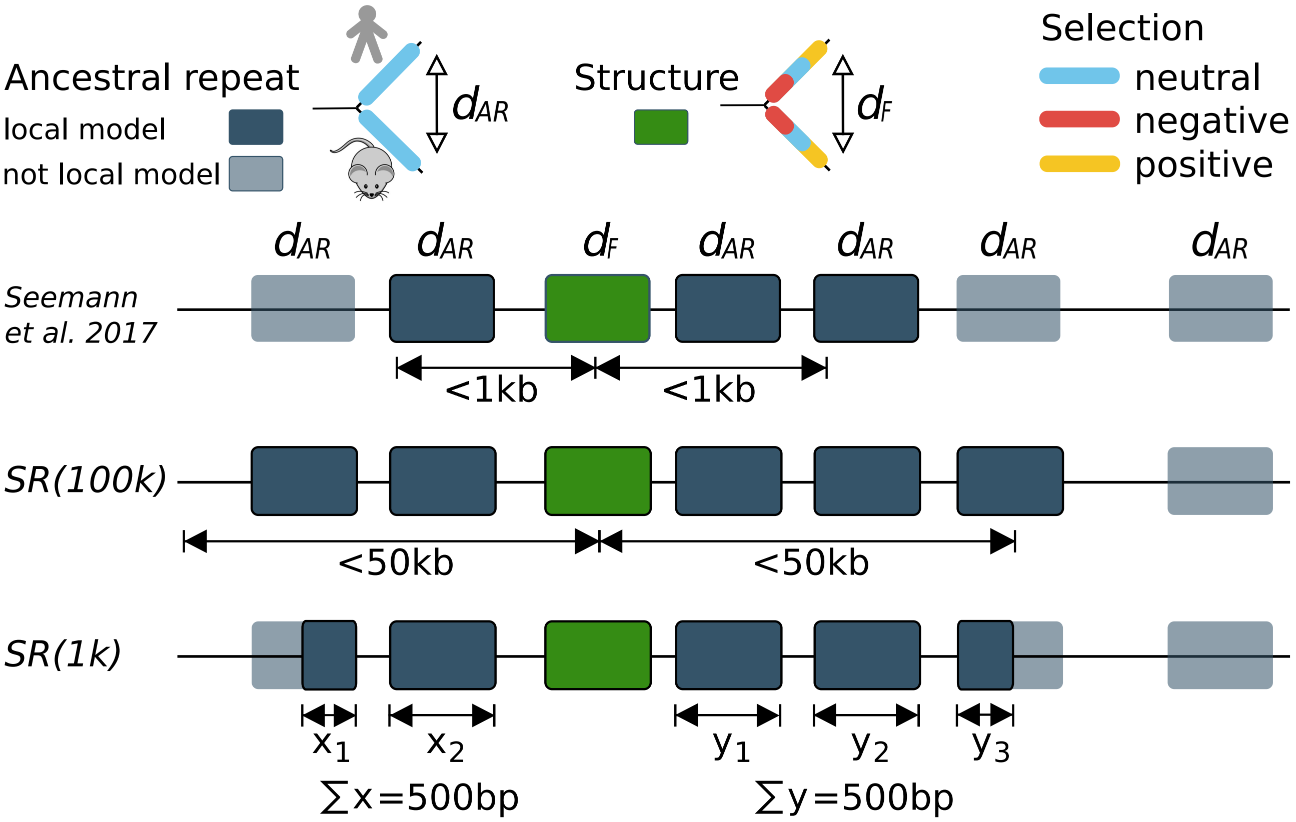

Figure S1: Schematic description of three different local neutral models. *SR(1k)* and *SR(100k)* have been tested in this study.

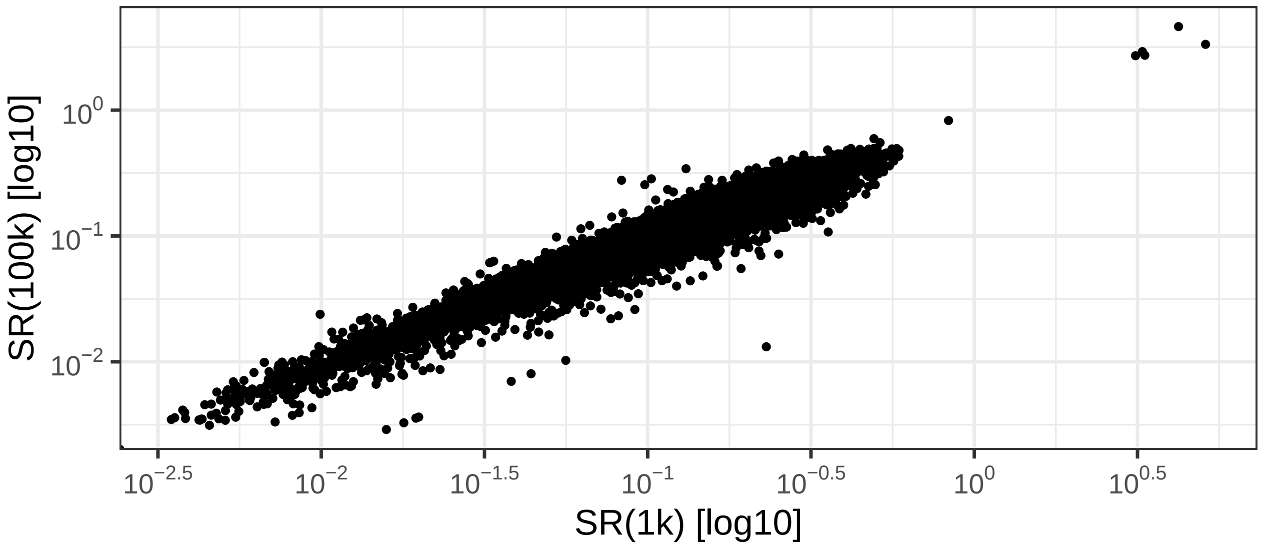

Figure S2: Scatter plot of selection ratios *SR*s of CRSs with both FDR(*SR(1k)*) and FDR(*SR(100k)*) being less or equal than 0.2. The Pearson's correlation coefficient ρ is 0.94. After removing the 5 outliers (both *SR(1k)* and *SR(100k)* greater than 2) $\rho = 0.93$. One of the 10 independent samplings of ARs is shown.

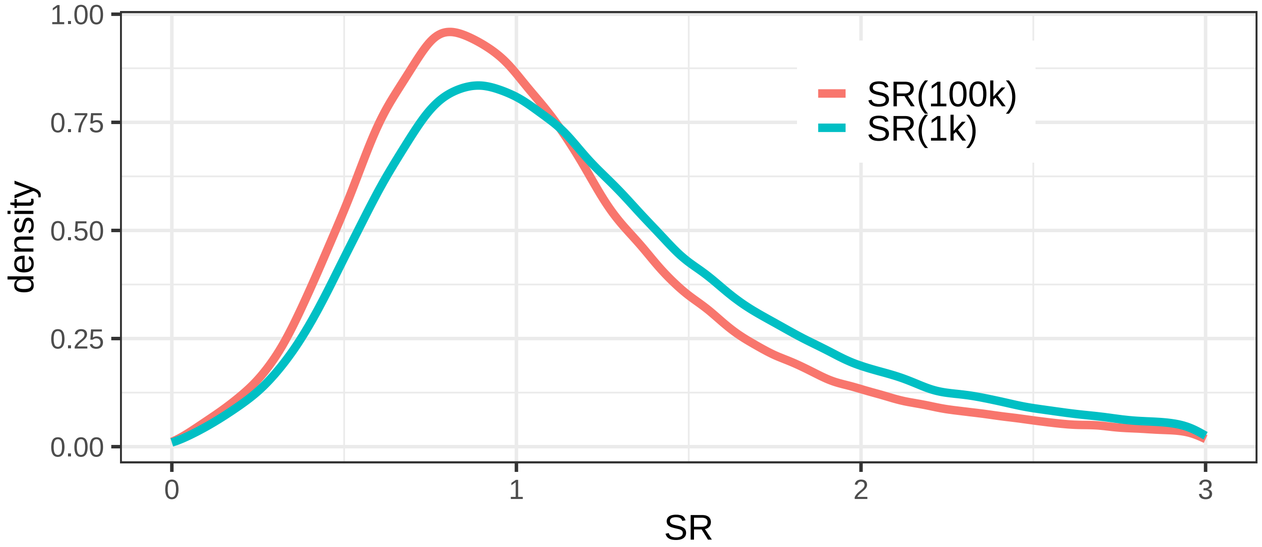

Figure S3: The distribution of selection ratio *SR* for two types of local neutral models. As expected, the *SR* of ancestral repeats is close to one. We decided to base our study on *SR(1k)* as its distribution is more centered around 1.0. One of the 10 independent samplings of ARs is shown.

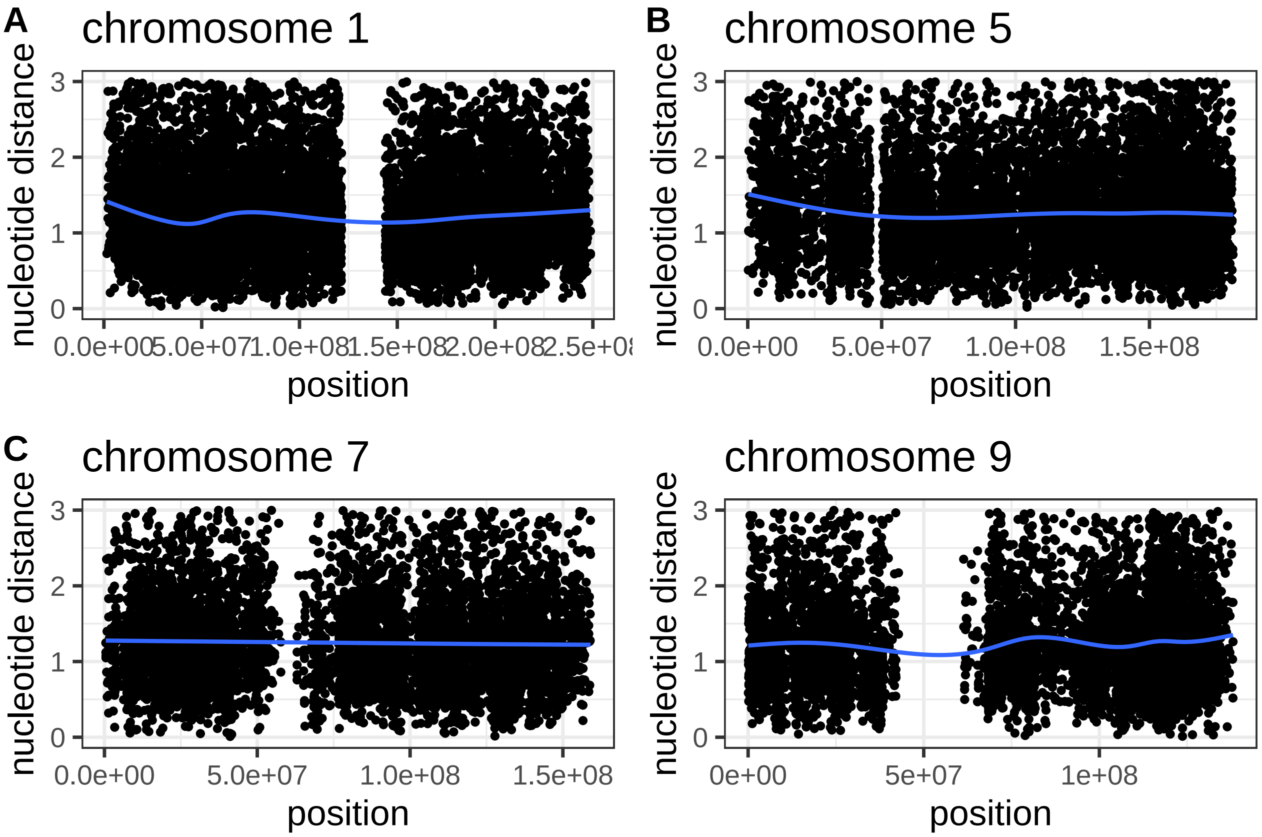

Figure S4: Human-mouse nucleotide distance of ancestral repeats (ARs) in four different human chromosomes. Pairwise nucleotide distance is calculated by baseml with REV/GTR model. A generalized additive model (GAM) with restricted maximum likelihood (REML) parameter estimation is fitted to the data (blue curves). One of the 10 independent samplings of ARs is shown. Whereas in chromosome 7 the mutation rate of ARs does not vary much, in the other three chromosomes it varies in the mega-base scale.

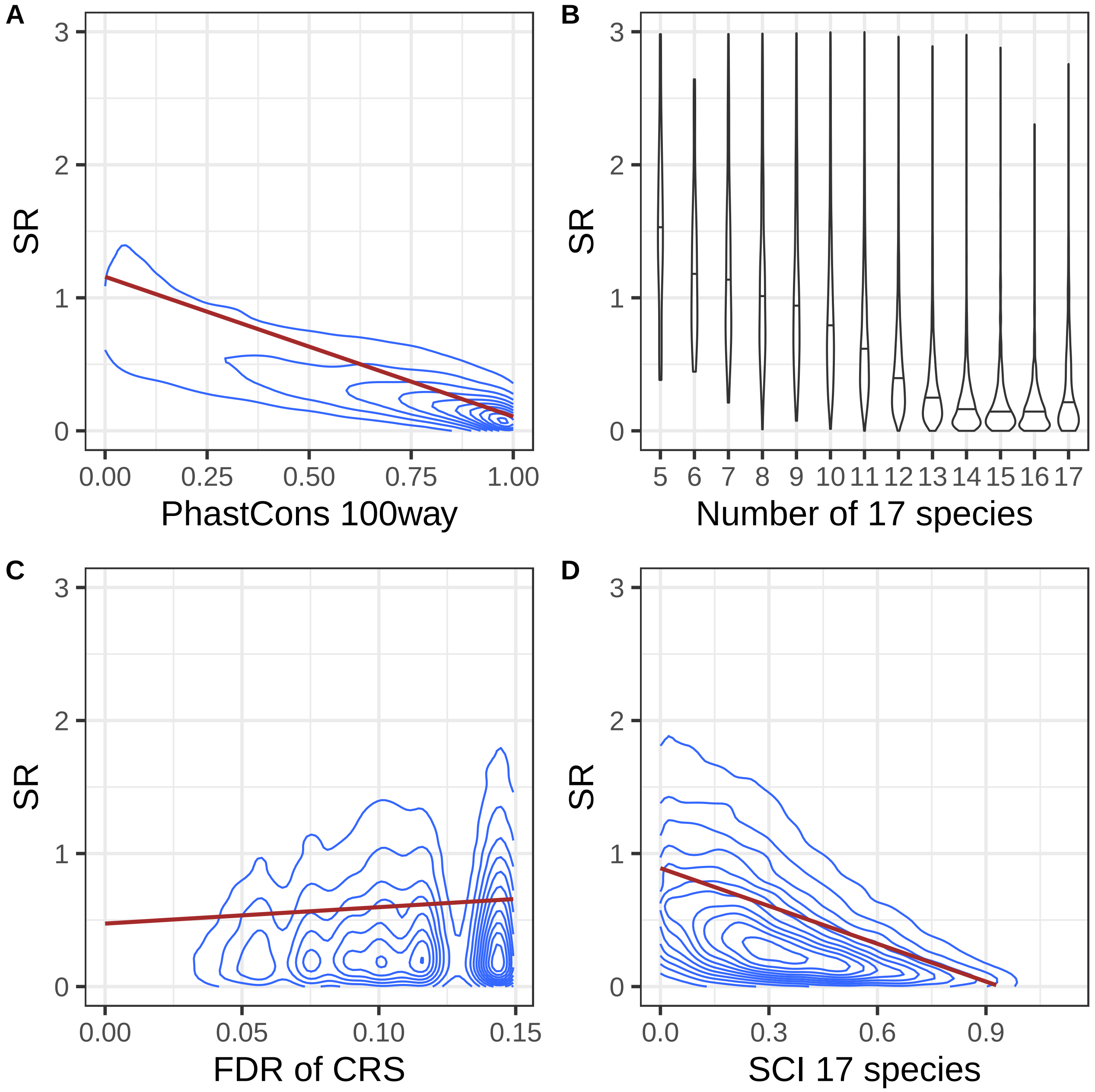

Figure S5: Correlation between selection ratio SR and different features of CRSs. **A** PhastCons scores originating from the 100 species UCSC alignments overlapping the human sequence of CRSs. **B** Number of sequences in the 17 species structure alignment (violin plot). **C** False Discovery rate (FDR) of conserved RNA structure (CRS) prediction. **D** Structure Conservation Index (SCI) computed on the 17 species structure alignment. The 2d density estimation and the linear regression are shown in **A**, **C** and **D**. Only SR lower than 3 is shown.

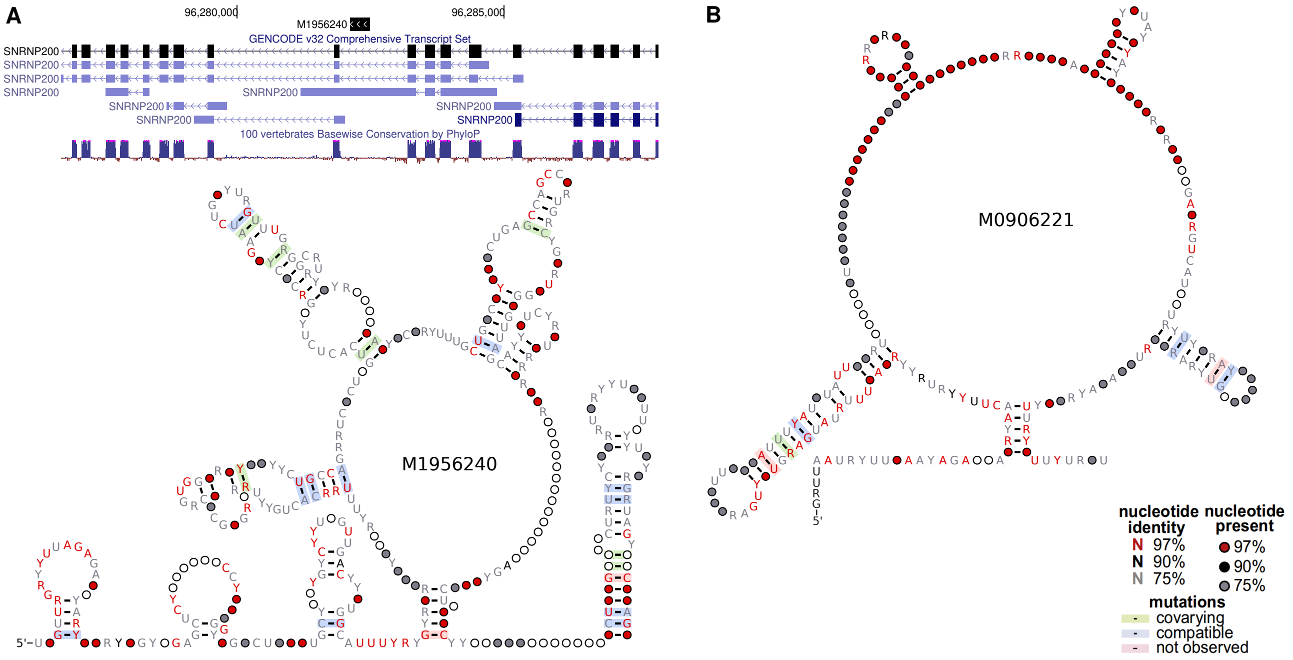

Figure S6: Examples of *de novo* structures with rapidly evolving sequence. Conservation patterns indicated in RNA secondary structures are based on 17 species structure based alignments (drawing by R2R [8]). **A** *M1956240* is located in a retained intron of SNRNP200 (hg38/chr2:96282115-96282488) and has the following properties: *SR*=2.4, *FDR(SR)*=0.18, GC(human)=0.48, SI(17 species)=55.92%, Length(17 species)=417 bp, SCI(17 species)=0.11. The corresponding protein is a core component of the spliceosome that catalyzes an ATP-dependent unwinding of U4/U6 RNA duplexes. The noncoding isoform is highly conserved in its overlapping exons of SNRNP200 but its sequence evolved neutrally in the remaining loci (see PhyloP track). **B** *M0906221* is intronic of the mRNA CAMK4 (hg38/chr5:111287401-111287622) and has the following properties: *SR*=3.3, *FDR(SR)*=0.06, GC(human)=0.25, SI(17 species)=55.23%, Length(17 species)=241 bp, SCI(17 species)=0.19.

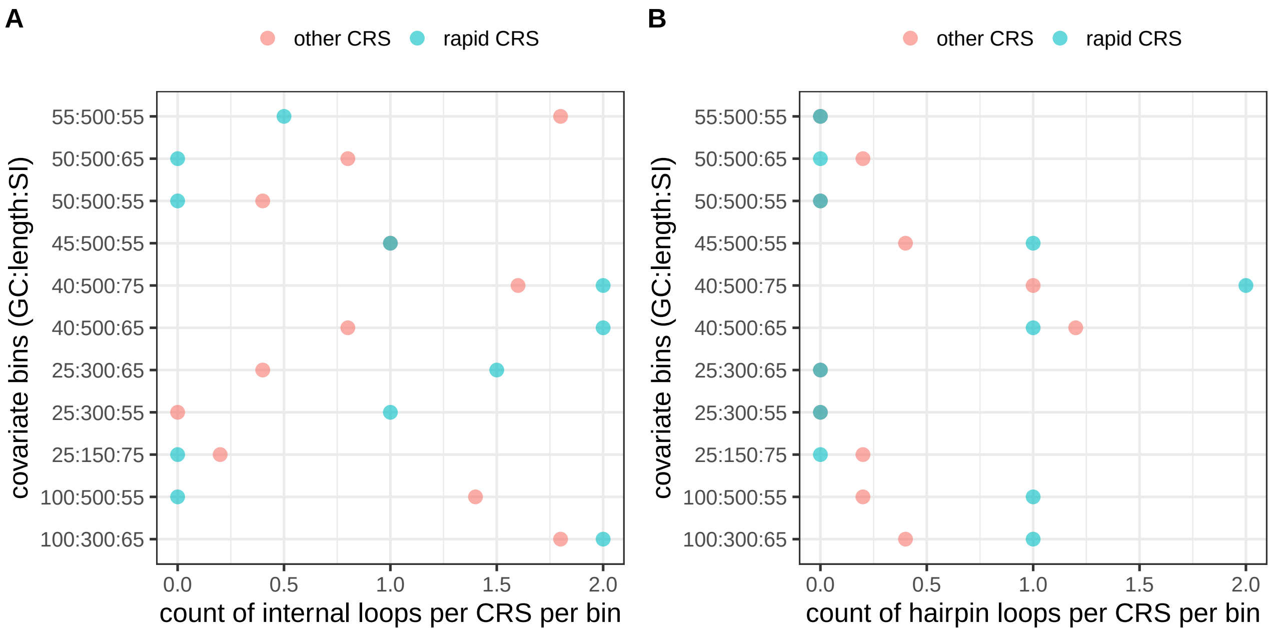

Figure S7: Observed and expected counts of **A** internal and **B** hairpin loop RNA 3D motifs. Observed are the 13 CRSs with rapidly evolving sequence. They can be grouped into 11 covariate bins of G+C content in human (%), length of the 17 species alignment, and sequence identity (SI) of the 17 species alignment (%) (y-axis). The expected counts are estimated from five representative CRSs in each of the 11 covariate bins. For each bin the upper boundary of each covariate is indicated in the labelling of the y-axis. The ranges are for G+C content [0-25],(25-30],(30-35],(35-40],(40-45],(45-50],(50-55],(55-60],(60-100]; for length (0-100],(100-150],(150-200],(200-300],(300-500]; and for SI (0-55],(55-65],(65-75],(75-85],(85-100]. The RNA 3D motifs are from the RNA 3D Motif Atlas and were fitted into loop sequences of CRSs and filtered as described in the Materials and Methods section.

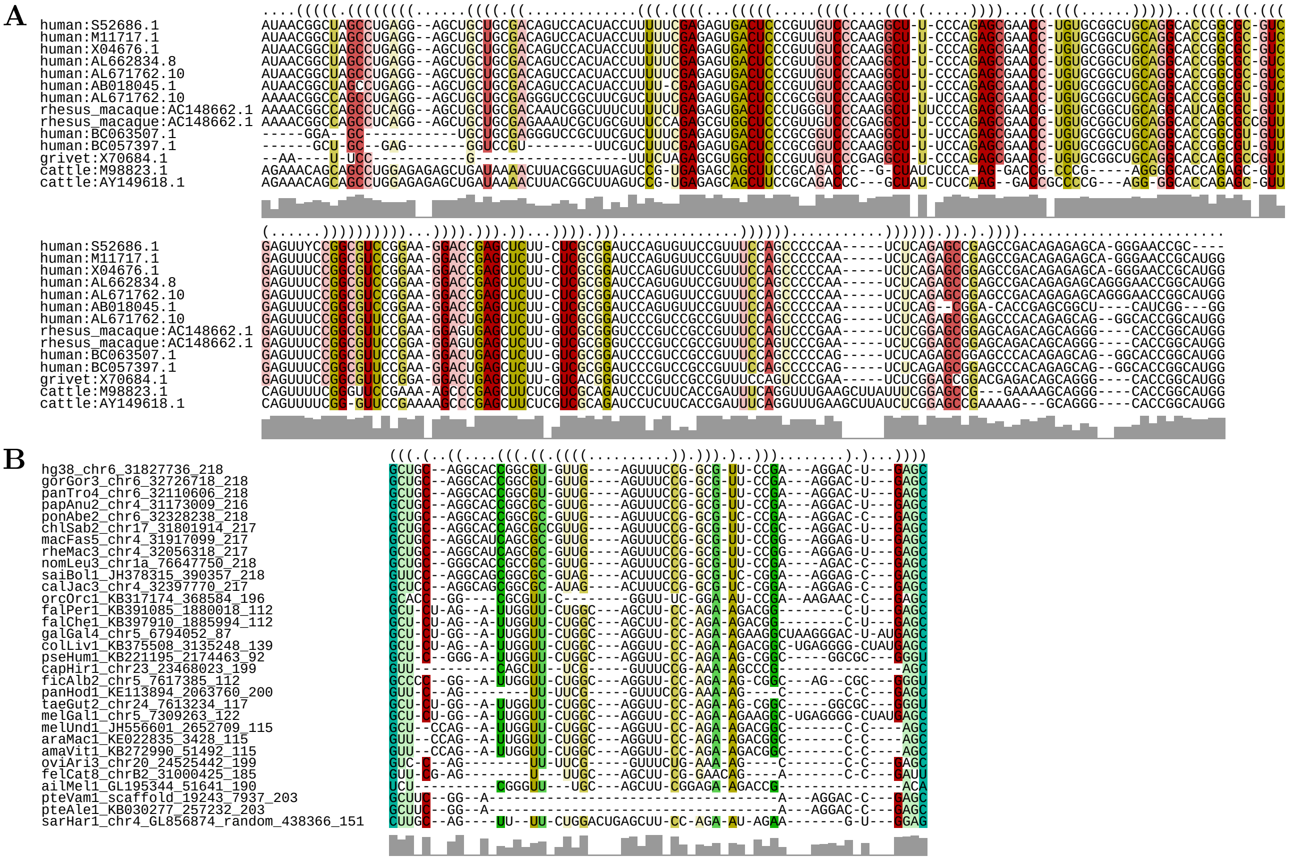

Figure S8: Rfam seed and CMfinder predicted alignment of the Rfam family with highest sequence divergence between human and mouse: *IRES Hsp70*. **A** Rfam (version 14.0) seed alignment consists of 12 sequences from primates and 2 from cattle. **B** CMfinder (version 0.2.2) predicted structure-based alignment of highest pscore (pscore=101). Despite mouse (mm10) being part of the input human (hg38) centered 100-way vertebrate UCSC Genome Browser alignment block overlapping human sequences from the Rfam seed alignment, the mouse sequence is not included in the CMfinder predicted motif. The color code represents the level of basepair conservation (http://rna.tbi.univie.ac.at/RNAWebSuite/help.html#A8) and barplot the sequence conservation.

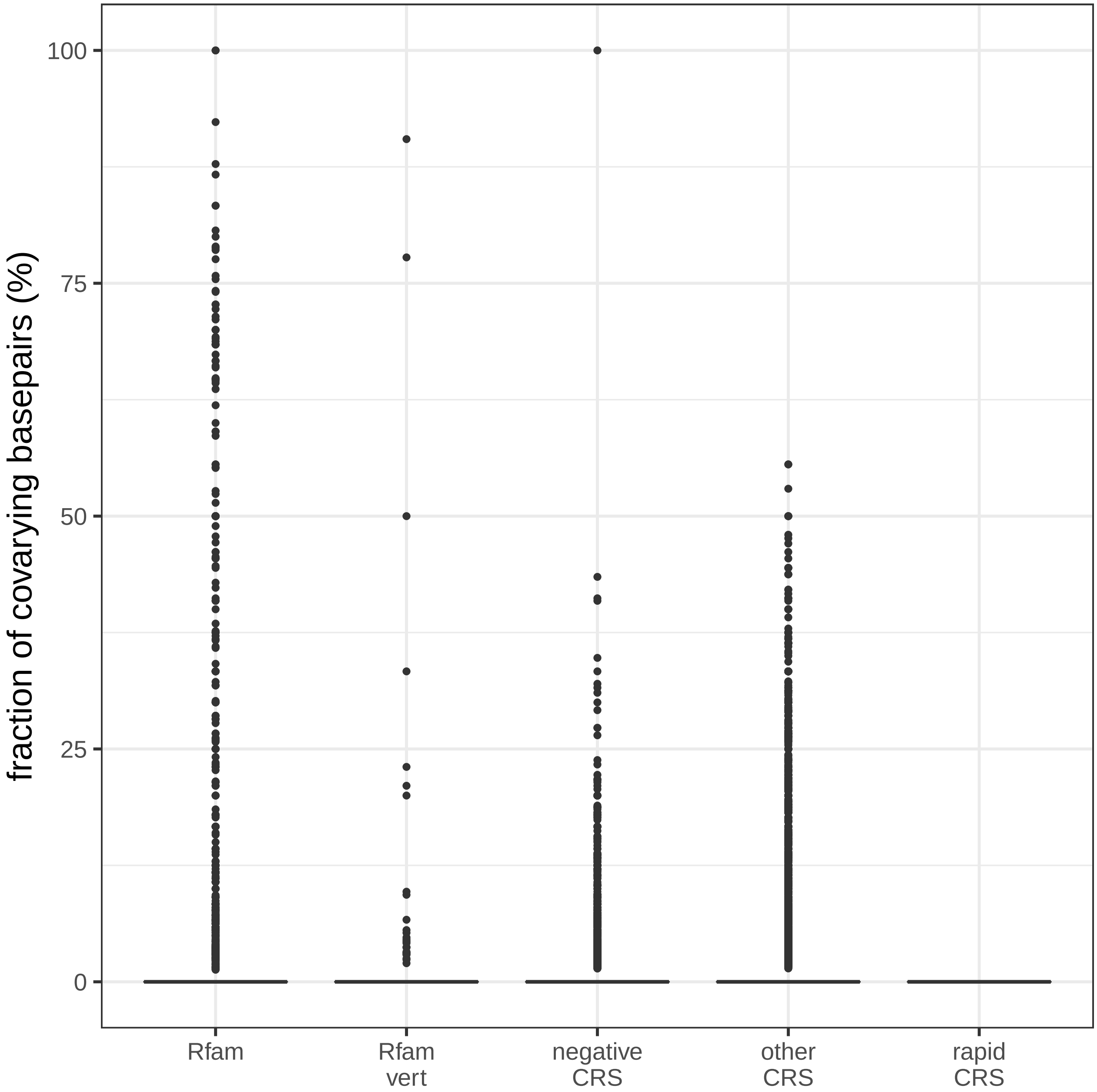

Figure S9: Fraction of basepairs that show a significant covariation signal in the one-set statistical test (all pairs are tested as equivalent) by R-scape (E<0.05). We distinguish CRSs with rapidly evolving sequence (*rapid CRS*: *SR*>2 and *FDR(SR)*≤0.2), CRSs under negative selection (*negative CRS*: *SR*<0.5 and *FDR(SR)*≤0.2), and other CRSs (*other CRS*). *Rfam* comprises 2,021 seed alignments of 2,791 in Rfam version 14.0, and *Rfam vert* is the subset of 715 vertebrate sequence filtered seed alignments. For the others (including *mir-657*) the covariation in the alignment is too small (mostly due to too few sequences). The observed number of covarying basepairs is zero for Rfam families with rapidly evolving sequences *IRES_Hsp70* and *FN_gamma*.

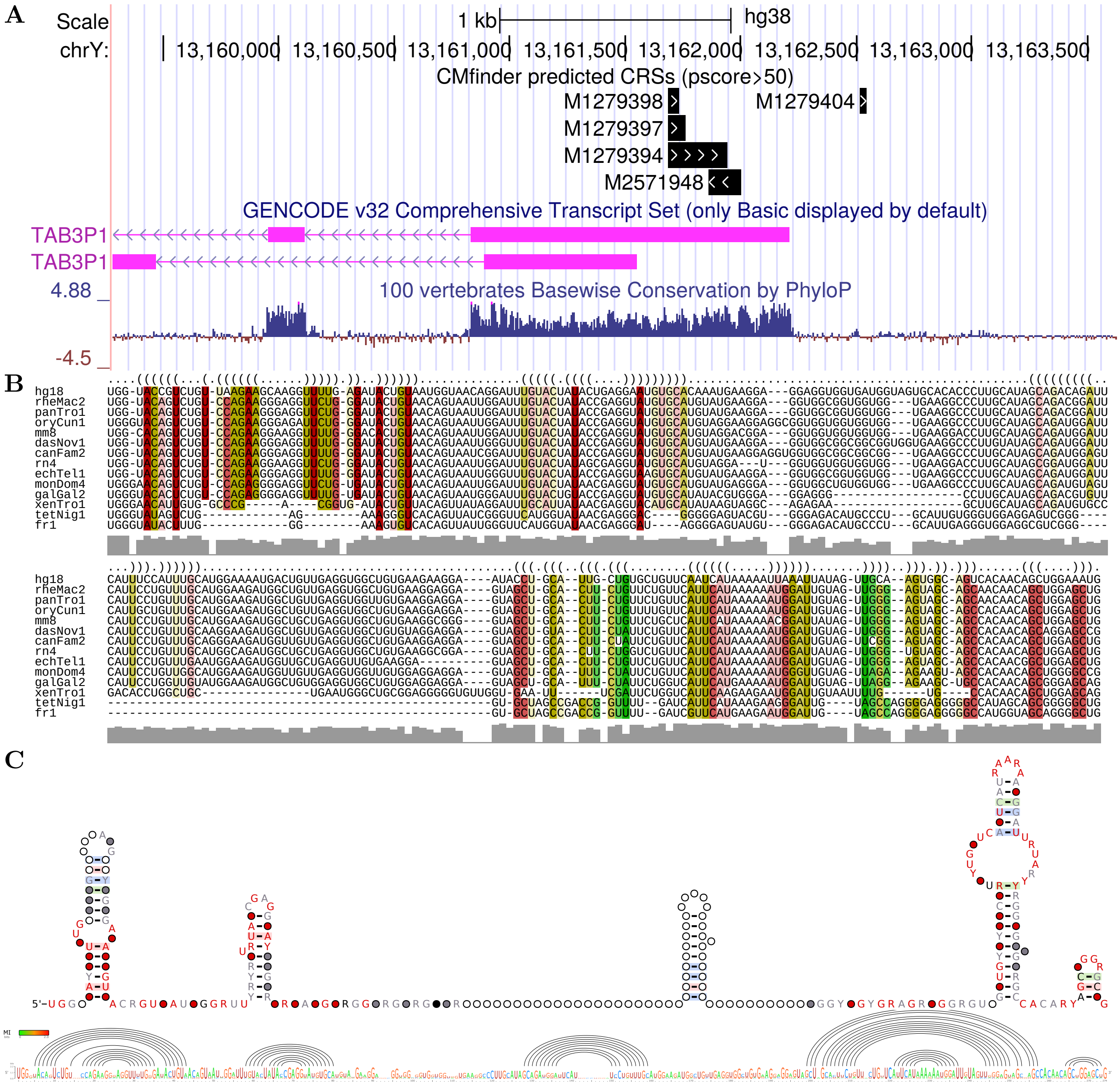

Figure S10: Human lineage specific CRS *M1279394*. *M1279394* is located at chrY:13161686-13161942 (hg38) and has the following properties: *SR*=0.35, *FDR(SR)*=0.05, SSS(human)=11.20, SSS(mouse)=7.00, SSS(macaque)=4.54, GC(human)=0.41, SI(17 species)=67.4%, length(17 species)=274 bp. **A** UCSC Genome Browser annotation tracks; **B** 17 species structure-based alignment predicted by `CMfinder` with color code representing the level of basepair conservation and barplot the sequence conservation; **C** RNA secondary structure predicted by `CMfinder` (drawing by R2R and RILogo using MI measure treeMI W+P [9]). There are no matching RNA 3D motifs predicted by `JAR3D`.

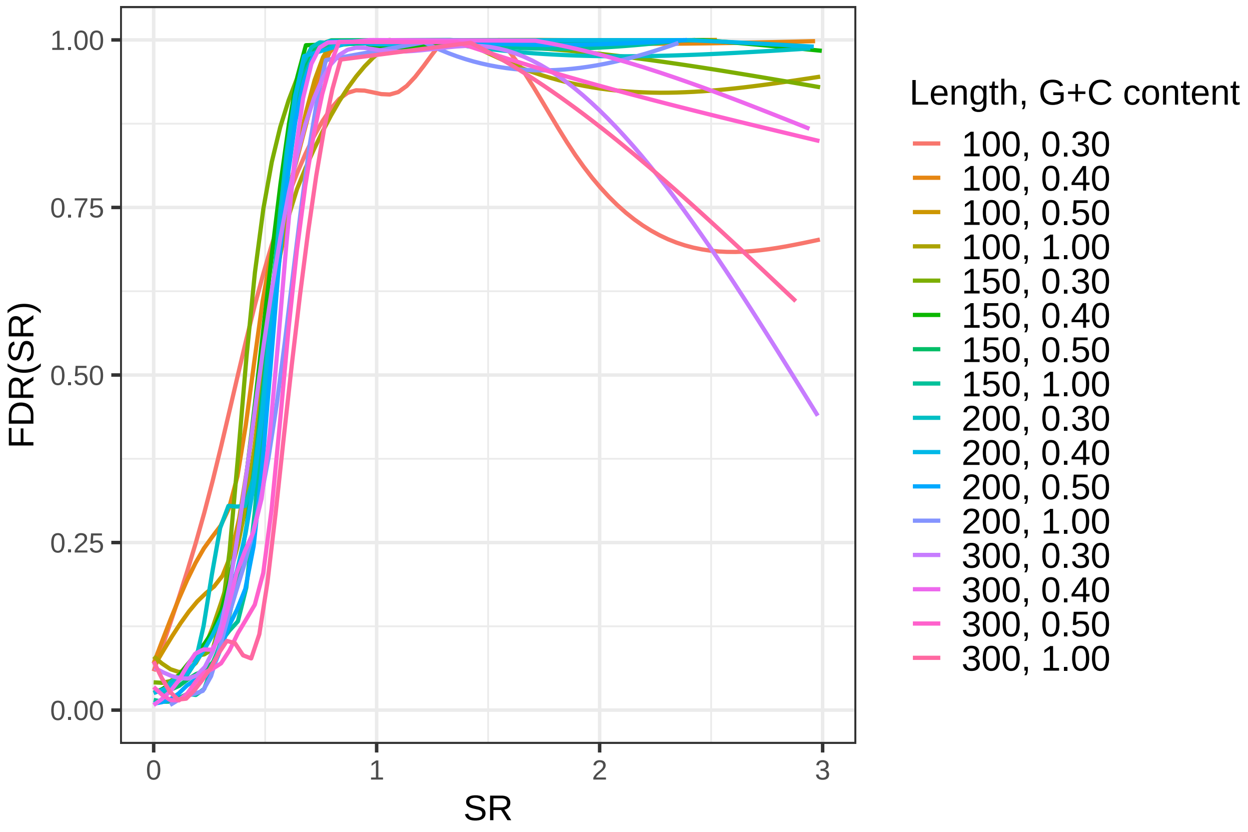

Figure S11: False discovery rate of the selection ratio, *i.e. FDR(SR)*, estimation of *de novo* structures. Structures and sampled ancestral repeats (null model of neutral selection) were divided into ranges of two covariates: "de-gapped" human-macaque-mouse alignment length [bp] (0-100],(100-150],(150-200],(200-300],(300-500], and human G+C content [0-0.25],(0.25-0.30],(0.30-0.35],(0.35-0.40],(0.40-0.45],(0.45-0.50],(0.50-0.55],(0.55-0.60],(0.60-1.00]. Here, some bins were merged for better visualization. All pairwise combinations of length and G+C content ranges were applied for *FDR(SR)* estimation. A generalized additive model (GAM) with restricted maximum likelihood (REML) parameter estimation is fitted to the data in each covariate range. One of the 10 independent samplings of ARs is shown. Only *SR*s lower than 3 are shown.

References

[1] Seemann, S., Mirza, A., Hansen, C., Bang-Berthelsen, C., Garde, C., Christensen-Dalsgaard, M., Torarinsson, E., Yao, Z., Workman, C., Pociot, F., Nielsen, H., Tommerup, N., Ruzzo, W., and Gorodkin, J. (Aug, 2017) The identification and functional annotation of RNA structures conserved in vertebrates. *Genome Res,* **27**(8), 1371–1383.

[2] Felsenstein, J. (2003) Inferring Phylogenies., Sinauer Associates, Sunderland, MA.

[3] Yang, Z. (Aug, 2007) PAML 4: phylogenetic analysis by maximum likelihood.. *Mol Biol Evol,* **24**(8), 1586–1591.

[4] Sweeney, B., Roy, P., and Leontis, N. (Jan, 2015) An introduction to recurrent nucleotide interactions in RNA. *Wiley Interdiscip Rev RNA,* **6**(1), 17–45.

[5] Petrov, A., Zirbel, C., and Leontis, N. (Oct, 2013) Automated classification of RNA 3D motifs and the RNA 3D Motif Atlas. *RNA,* **19**(10), 1327–1340.

[6] Zirbel, C., Roll, J., Sweeney, B., Petrov, A., Pirrung, M., and Leontis, N. (Sep, 2015) Identifying novel sequence variants of RNA 3D motifs. *Nucleic Acids Res,* **43**(15), 7504–7520.

[7] Walter Costa, M., Honer Zu Siederdissen, C., Dunjic, M., Stadler, P., and Nowick, K. (Mar, 2019) SSS-test: a novel test for detecting positive selection on RNA secondary structure. *BMC Bioinformatics,* **20**(1), 151.

[8] Weinberg, Z. and Breaker, R. (Jan, 2011) R2R–software to speed the depiction of aesthetic consensus RNA secondary structures. *BMC Bioinformatics,* **12**, 3.

[9] Menzel, P., Seemann, S., and Gorodkin, J. (Oct, 2012) RILogo: visualizing RNA-RNA interactions. *Bioinformatics,* **28**(19), 2523–2526.
